# Multiomic integration reveals neuronal‐extracellular vesicle coordination of gliotic responses in degeneration

**DOI:** 10.1002/jev2.12393

**Published:** 2023-12-11

**Authors:** Adrian V. Cioanca, Yvette Wooff, Riemke Aggio‐Bruce, Rakshanya Sekar, Catherine Dietrich, Riccardo Natoli

**Affiliations:** ^1^ Clear Vision Research Group, Eccles Institute of Neuroscience, John Curtin School of Medical Research, College of Health and Medicine The Australian National University Canberra Australia; ^2^ School of Medicine and Psychology, College of Health and Medicine The Australian National University Canberra Australia; ^3^ Peter MacCallum Cancer Centre Melbourne Victoria Australia

**Keywords:** central nervous system, extracellular vesicles, glia, neurodegeneration, retina, tissue‐cross talk

## Abstract

In the central nervous system (CNS), including in the retina, neuronal‐to‐glial communication is critical for maintaining tissue homeostasis including signal transmission, transfer of trophic factors, and in the modulation of inflammation. Extracellular vesicle (EV)‐mediated transport of molecular messages to regulate these processes has been suggested as a mechanism by which bidirectional communication between neuronal and glial cells can occur. In this work we employed multiomics integration to investigate the role of EV communication pathways from neurons to glial cells within the CNS, using the mouse retina as a readily accessible representative CNS tissue. Further, using a well‐established model of degeneration, we aimed to uncover how dysregulation of homeostatic messaging between neurons and glia via EV can result in retinal and neurodegenerative diseases. EV proteomics, glia microRNA (miRNA) Open Array and small RNA sequencing, and retinal single cell sequencing were performed, with datasets integrated and analysed computationally. Results demonstrated that exogenous transfer of neuronal miRNA to glial cells was mediated by EV and occurred as a targeted response during degeneration to modulate gliotic inflammation. Taken together, our results support a model of neuronal‐to‐glial communication via EV, which could be harnessed for therapeutic targeting to slow the progression of retinal‐, and neuro‐degenerations of the CNS.

## 1 INTRODUCTION

Neurodegeneration and gliosis are intricately interconnected, playing an important role in the development and progression of diseases affecting the CNS. Gliosis, characterized by the activation of specific glial cells such as astrocytes in the brain, Müller glia in the retina, and microglia in both, is a prominent pathological feature contributing to various retinal and neurodegenerative diseases including age‐related macular degeneration, Alzheimer's and Parkinson's diseases and multiple sclerosis (Bringmann et al., 2006; Leyns and Holtzman, 2017; Pekny & Pekna, 2016). Under normal physiological conditions, glial cells provide trophic and metabolic support to all neurons including photoreceptors, the light‐sensing cells of the retina (Bringmann et al., 2006; Newman & Reichenbach, 1996; Stevenson et al., 2020). However, in degenerative diseases, these cells can become gliotic and contribute to the inflammatory milieu (Bringmann et al., 2006; Newman & Reichenbach, 1996; Stevenson et al., 2020). While early or reactive gliotic responses aim to limit damage and restore homeostasis, hallmark features of proliferative (pathological) gliosis include the upregulation of intermediate filament proteins such as glial fibrillary acidic protein (GFAP) and vimentin (VIM), cell hypertrophy, and the release of proinflammatory mediators resulting in a loss of homeostasis and exacerbation of degeneration (Bringmann et al., 2009, 2006; Kauppinen et al., 2016; Rutar et al., 2015).

As a simplified and representative tissue of the CNS, the retina is a valuable and translatable model for investigating neuronal‐to‐glial communication pathways, particularly in the context of CNS diseases where gliosis plays a significant role. Our recent observations revealed that localized neuronal damage in the retina elicited a rapid gliotic response that not only preceded neuronal apoptosis but also extended considerably beyond the affected region (Wooff et al., 2023). Intriguingly, this phenomenon is similarly observed in other models of localized injury, such as in retinal detachment (Lee et al., 2021) and in traumatic brain injury (Szepesi et al., 2018). These observations advocate for the presence of an underlying signal propagation mechanism by which neuronal cells are able to disseminate danger signals far from the site of damage. However, to date, remarkably few communication models have been put forth to adequately explain how neurons, including photoreceptors, can communicate and influence glia. Understanding how gliosis is regulated and propagated is therefore essential in the development of therapeutic strategies to prevent the progression of retinal and neurodegenerations.

Recent evidence suggests that extracellular vesicle (EV) communication axes exist within the retina (Flores‐Bellver et al., 2021; Wooff et al., 2020; Zhang et al., 2021), and brain (Ahmad et al., 2022; Schnatz et al., 2021), and functions to mediate neuronal‐to‐glial crosstalk (Ahmad et al., 2022; Kalargyrou et al., 2021; Ogaki et al., 2021; Schnatz et al., 2021; Wooff et al., 2020). EV are nanosized endogenous cell‐to‐cell communication vehicles that selectively encapsulate and transport molecular cargo including DNA, proteins and regulatory RNA such as microRNA (miRNA), to shape the biology of recipient cells (Isola & Chen, 2017; Mathieu et al., 2019; Yuana et al., 2013). Work by Kalargyrou et al. (2021) demonstrated that in the healthy retina, photoreceptors endogenously released EV to selectively target and communicate with Müller glia (Kalargyrou et al., 2021), while Demais et al. (2022) also reported a strong Müller‐derived contribution of EV in the retina (Demais et al., 2022). In the brain, bidirectional transfer of EV cargo between neurons and astrocytes has also been shown to influence neuronal morphology and synaptic homeostasis, as well as modulate gliotic states (Ahmad et al., 2022; Ogaki et al., 2021; Schnatz et al., 2021). In particular, EV transport of miRNA between neurons and glia has been well studied in the CNS, and in the pathogenesis of neurodegenerative disorders (Antoniou et al., 2023; Ogaki et al., 2021; Wooff et al., 2020). In fact, findings from our group highlighted that EV transport of a neuronal miRNA, miR‐124‐3p, between photoreceptors and Müller glia was required for homeostatic regulation, with disruption of EV communication not only reducing miR‐124‐3p trafficking along this axis but consequently exacerbated retinal degeneration (Wooff et al., 2020). Additional mechanistic studies performed by our group established that miR‐124‐3p was able to undergo a motif shift in response to degeneration that biased its targetome towards mRNA associated with the establishment of gliosis—specifically *Ccl2*, a known chemokine implicated in retinal (Chu‐Tan et al., 2021, 2018; Rutar et al., 2011) and neurodegenerations (Joly‐Amado et al., 2020). Outside of the visual system, a similar EV‐dependent communication axis was identified in the brain, where miR‐124‐3p transported in EV between neurons and astrocytes was shown to be involved in regulating glutamate uptake (Men et al., 2019) and influencing phenotypic changes in microglia (Ponomarev et al., 2011; Veremeyko et al., 2019). While EV appear central to material exchanges between neurons and glia; the regulators of EV tropism and the extent and functional consequences of miRNA cargo trafficked between neurons and glia in the context of the CNS, remains unknown.

In the current study, we therefore examined the capacity of EV to mediate neuronal‐to‐glia communication using the retina as a model CNS tissue, and how alterations in this communication pathway may modulate gliotic responses in degenerations. Key results from this study identified that retinal EV had altered proteomic signatures in response to retinal degeneration and highlighted potential protein interactions underlying the tropism of retinal EV towards Müller glia and microglia. Subsequently, we showed that in degenerating photoreceptors, alterations in the EV proteome were associated with the dysregulation of EV biogenesis, trafficking, and miRNA loading processes. Lastly, by exploring the miRNA content of retinal EV and glia, we identified a trio of miRNAs (miR‐183/96/182) as selective RNA cargo exchanged between photoreceptors and Müller glia during degeneration and described the regulatory networks of these miRNAs as key instigators in early gliotic responses in disease. These findings together strengthen our understanding of EV tropism to glia during both normal neuronal health and in degenerative states and may be more broadly relevant for a wide variety of neurodegenerative diseases.

## 2 MATERIALS AND METHODS

### 2.1 Animal paradigms

#### 2.1.1 Animal handling

All experiments were conducted in accordance with the ARVO Statement for the Use of Animals in Ophthalmic and Vision Research and with approval from the Australian National University's (ANU) Animal Experimentation Ethics Committee (AEEC) (Ethics ID: A2020/41; Rodent models and treatments for retinal degenerations). For EV experiments, adult male and female C57BL/6J wild‐type (WT) mice (aged 60 postnatal days; (P50) at experimental onset) were purchased from the Animal Resources Centre (ARC), (Canning Vale, Western Australia (WA)). Adult PDGFRa‐cre (platelet derived growth factor receptor, alpha polypeptide) mice (animal #013148; The Jackson Laboratory, Bar Harbor, ME, USA) were used for Müller cell isolation either in control/dim‐reared (DR) conditions or after photo‐oxidative damage (PD). PDGFRa‐cre animals were crossed with reporter strain B6.Cg‐Gt(ROSA)26Sortm14 (animal #007914; The Jackson Laboratory) to produce PDGFRa‐cre RFP mice, which were maintained on a C57BL/6J genetic background as described previously (Chu‐Tan et al., 2018). All strains were maintained on the C57BL/6J genetic background and screened for the Rpe65Leu polymorphism or the deleterious *Crb1/Rd8* mutation prior to crossing using previously validated PCR primers (Kim et al., 2004; Mattapallil et al., 2012). Mice were bred, reared, and housed under 12 h light/dark cycle conditions (5 lux) with free access to food and water.

#### 2.1.2 Photo‐oxidative damage

As a model of retinal degeneration C57BL/6J or PDGFRa‐cre RFP mice were subjected to photo‐oxidative damage (PD) for 5 days as described previously (Natoli et al., 2016). This model recapitulates key disease features common to retinal and neurodegenerative diseases including functional losses, neuronal cell death, inflammatory propagation, microglial recruitment and activation and gliosis (Natoli et al., 2016). Briefly, mice were placed into Perspex boxes coated with a reflective interior surface and exposed to 100 K lux white light from light‐emitting diodes (LED). Animals were administered pupil dilator (Minims® atropine sulphate 1% w/v; Bausch and Lomb) to both eyes twice a day (9 AM and 4 PM) during the course of the damage paradigm. Mice were euthanized with CO_2_ following experimental runs. Eyes from dim‐reared (DR; five lux light) control and PD PDGFRa mice were collected for cryosectioning and histological analyses, while retinas from C57BL/6J mice were collected for extracellular vesicle (EV) isolation.

### 2.2 Retinal assessment

#### 2.2.1 Retinal tissue collection and preparation

Animals were euthanized with CO_2_ following experimental paradigms. The superior surface of the left eye was marked and enucleated, then immersed in 4% paraformaldehyde for 3 h. Eyes were then cryopreserved in 15% sucrose solution overnight, embedded in OCT medium (Tissue Tek, Sakura, Japan) and cryosectioned at 12 μm in a parasagittal plane (superior to inferior) using a CM 1850 Cryostat (Leica Biosystems, Germany). To ensure accurate comparisons were made for histological analysis, only sections containing the optic nerve head were used for analysis. The retina from the right eye was excised through a corneal incision and placed into RNAlater solution (Thermo Fisher Scientific, MA, United States) at 4°C overnight and then stored at −80°C until further use.

#### 2.2.2 Immunohistochemistry

Immunolabeling for Glial Fibrillary Acidic Protein (GFAP) (1:500, ASTR06, MA5‐12023, Invitrogen, Massachusetts, USA), a marker of glial cell stress, and Glutamine synthase (GLUL,1:1000, ab197024, Abcam, Cambridge, UK), a Müller cell marker, was performed as previously described (Rutar et al., 2015). Retinal cryosections were stained with the DNA‐specific dye bisbenzimide (1:10000, Sigma‐Aldrich, MO, United States) to visualize the cellular layers.

#### 2.2.3 TUNEL assay

Terminal deoxynucleotidyl transferase (Tdt) dUTP nick end labelling (TUNEL), was used as a measure of photoreceptor cell death. TUNEL in situ labelling was performed on retinal cryosections using a Tdt enzyme (Cat# 3333566001, Sigma‐Aldrich, MO, United States) and biotinylated deoxyuridine triphosphate (dUTP) (Cat# 11093070910, Sigma‐Aldrich, MO, United States) as previously described (Natoli et al., 2010). Images of TUNEL staining were captured with the A1^+^Nikon confocal microscope at 20 and 40× magnification. The total number of TUNEL^+^ cells were counted including both the superior and inferior retina using two retinal sections per animal. To further quantify photoreceptor survival, the thickness of the ONL on retinal cryosections was measured along retinal cryosections on comparable sections between groups.

Immunohistochemical analysis of retinal cryosections was performed as previously described (Rutar et al., 2015). Fluorescence was visualized and images taken using a laser‐scanning A1^+^confocal microscope at 20× magnification (Nikon, Tokyo, Japan). Images panels were analysed using ImageJ V2.0 software and assembled using Photoshop CS6 software (Adobe Systems, CA, United States).

### 2.3 Fluorescence‐assisted cell sorting of glia

Müller glia and microglia were FACS isolated as previously described (Chu‐Tan et al., 2018; Rutar et al., 2015). Briefly, retinas were extracted from DR and 5d PD PDGFRa‐Cre RFP reporter mice (four retinas per sample) and DR and 7d PD Cx3Cr1‐Cre GFP reporter mice (6; DR‐12; PD retinas per sample) using a corneal incision. Retinas were placed in ice‐cold Hanks buffered saline solution (HBSS, Gibco; Thermo Fisher Scientific, MA, USA). Following light dissection, retinal pieces were digested in 500 μL of papain solution ((HBSS with 2.5 mg/mL papain (Worthington Biochemical, NJ, USA), 200U DNAse I (Roche Diagnostics, NSW, AUS) 5 μg/mL catalase (Sigma‐Aldrich, MO, USA), 10 μg/mL gentamycin (Sigma‐Aldrich, MO, USA) and 5 μg/mL superoxide dismutase (Worthington Biochemical, NJ, USA)) for 8 min at 37°C and a further 15 min at 8°C with two rounds of light trituration (10 times, 1000 μL pipette tip) at the 5‐ and 10‐min mark. Following digestion, cells were centrifuged (300 *g*, 4°C, 5 min) then resuspended in a papain neutralization solution ((HBSS with 4% bovine serum albumin (BSA, Thermo Fisher, MA, USA), 50 μg/mL antipain dihydrochloride (Roche Diagnostics, NSW, AUS), 200U DNAse I (Roche Diagnostics, NSW, AUS) 5 μg/mL catalase (Sigma‐Aldrich, MO, USA), 10 μg/mL gentamycin (Sigma‐Aldrich, NSW, AUS) and 5 μg/mL superoxide dismutase (Worthington Biochemical, NJ, USA)) for 10 min at 4°C. Next, the cells were passed through a 70 μm SmartStrainers filter mesh (Miltenyi Biotec, Cologne, Germany), washed twice in cell sorting buffer (HBSS with 4% bovine serum albumin (BSA, Thermo Fisher, MA, USA), 200U DNAse I (Roche Diagnostics, NSW, AUS) then submitted for cell sorting at the JCSMR Imaging and Flow facility (Australian National University).

Cells were sorted using a FACSAria II (BD Biosciences, Franklin Lakes, NJ, USA), gating for RFP+ cells or GFP+ cells using a standard gating strategy combining side versus forward scatter, side scatter width versus height, and DAPI or Trypan Blue staining for viability to discriminate viable single cells events from cell debris and doublets. Isolated cells were processed for RNA isolation with miRVana RNA isolation kit (Thermo Fisher Scientific, MA, USA) immediately after sorting.

The viability and Müller cell authenticity of RFP^Hi^ cells was determined by post sort DAPI staining and immunolabelling with Glutamine synthase (GLUL, 1:1000, ab197024, Abcam, Cambridge, UK). Prior to immunolabelling, of RFP^Hi^ cells were fixed in 2% paraformaldehyde for 10 min, then permeabilized in 0.05% TritonX for 5 min. After two rounds of washing in PBS, the cells were incubated in primary antibodies for 1 h, then secondary antibodies for 1 h. Following incubation, cell suspensions were washed twice in PBS then a 10 μL volume was placed on hemocytometer and imaged on a confocal microscope.

### 2.4 RNA isolation

Total RNA was isolated from FACS Müller glia and microglia using the miRVana Isolation Kit (Thermo Fisher Scientific, MA, USA) following manufacturers’ instructions. This combines an initial acid‐phenol:chloroform organic extraction followed by spin‐column purification and elution. The concentration and degradation of RNA was assessed using an Agilent Bioanalyzer with an Agilent Pico RNA assay (Agilent Technologies, CA, USA) and RNA was stored at −80°C until required.

### 2.5 cDNA, qPCR and analysis

Following purification of RNA, cDNA was synthesized from 1 μg RNA using the TaqMan MicroRNA RT kit (Thermo Fisher Scientific) from a miRNA template, according to manufacturers’ instructions. The expression of miRNA was measured using mouse specific TaqMan hydrolysis probes (Table 1) and TaqMan Gene Expression Master Mix (Thermo Fisher Scientific, MA, USA). Reactions were performed in technical duplicates in a 384‐well format using a QuantStudio 12 K Flex RT‐PCR machine (Thermo Fisher Scientific, MA, United States). Data was analysed using the comparative Ct method (ΔΔCt) and results are presented as percent change relative to control. Expression was normalized to reference gene small nuclear RNA U6 for miRNA.

**TABLE 1:** TaqMan hydrolysis probes (Thermo Fisher Scientific, MA, USA) used for qPCR.

Gene	Method	Primer sequence or TaqMan Assay ID
miR‐146‐5p	TaqMan	Hs04231522
miR‐223‐5p	TaqMan	002295
miR‐96‐5p	TaqMan	000186
miR‐182‐5p	TaqMan	002599
miR‐183‐5p	TaqMan	002269
miR‐124‐3p	TaqMan	001182
Pri‐miR‐96	SYBR	**F**:GTGCCAGGGTACAAAGACCT **R**:GGCACTACACATGATTGCTCA
Pri‐miR‐183	SYBR	**F**:TGTAGGACCTCCAGGAGAAGG **R**:TATGGCCCTTCGGTAATTCA
Pri‐miR‐182	SYBR	**F**:CCCTCCTAAAACCACCCTAA **R**:AGTTGGCAAGTCTAGAACCAC
Pri‐miR‐204	SYBR	**F**:GCTAAGATGCCGGAGAATCA **R**:GCCTTCCCAGCCTCCTTC
Pre‐miR‐96	TaqMan	Mm04238130
Pre‐miR‐183	TaqMan	Mm04934686
Pre‐miR‐182	TaqMan	Mm04238130
U6 snRNA	TaqMan	001973
18s RNA	SYBR	**F**:CGGCTACCACATCCAAGGAA **R**: GCTGGAATTACCGCGGCT
*Actb*	SYBR	**F**:TGTTACCAACTGGGACGACA **R**: GGAGAGCATAGCCCTCGTAG
*Apoe*	TaqMan	Mm01307193
*Gapdh*	TaqMan	Mm99999915_g1
*Glul*	TaqMan	Mm00725701

### 2.6 MicroRNA open‐array

#### 2.6.1 Preamplification and array card setup

To increase the sensitivity of the Open‐Array assay, cDNA from FACS Müller glia was first pre‐amplified before being profiled for miRNA. A total of 20 ng of RNA was as used as input in a 12 μL cDNA synthesis reaction separately containing MegaPlex microRNA primer pools A and B (Thermo Fisher Scientific, MA, USA) together screening for the presence of 754 well‐characterized mouse miRNAs. cDNA synthesis was performed using the following reaction mixture: 1 μL of MegaPlex miRNA primers, 0.3 μL 100 mM dNTP, 3 μL Multiscribe RT enzyme (50 U/μL), 1.5 μL 10°C ∼ RT buffer, 0.19 μL RNase inhibitor (20 U/μL). The synthesis consisted of a 30 min primer annealing 16°C, 30 min first‐strand synthesis at 42°C and 5 min at 85°C termination step. All reaction components are part of the TaqMan microRNA Reverse Transcription Kit (Thermo Fisher Scientific, MA, USA). Following cDNA synthesis, 3 μL of the RT reaction was diluted to 12.5 μL in water then mixed with 25 μL of TaqMan PreAmp Master Mix (Thermo Fisher Scientific, MA, USA) and 12.5 μL of PreAmp Primer pools (Thermo Fisher Scientific, MA, USA) then amplified using 12 PCR cycles. The PCR product was diluted 1:40, then 22.5 μL were mixed with 22.5 μL of TaqMan OpenArray Real‐Time PCR Master Mix (Thermo Fisher Scientific, MA, USA) and robotically loaded onto TaqMan OpenArray Human MicroRNA Panels (Thermo Fisher Scientific, MA, USA) using the AccuFill System (Thermo Fisher Scientific, MA, USA). The Open‐Array panels were cycled on the QuantStudio 12K Flex RT PCR system (Thermo Fisher Scientific, MA, USA) using 40 PCR cycles.

#### 2.6.2 Open‐array data analysis

Open‐array data processing was performed using the HTqPCR R/Bioconductor package. First, the relationship between raw Ct values and amplification scores (AmpScore) was examined using a 2‐dimensional density scatter plot to inform the selection of expression and amplification quality thresholds. Data points found either below the AmpScore threshold or Ct thresholds were labelled as ‘unreliable Cts’. MicroRNAs which had >3 unreliable Cts across the 12 samples profiled were excluded from further analysis. After filtering, using NormalizeCtData function three different normalization methods were applied based on rank‐invariant genes, scale invariant genes, and sample geometric means then the performance of each normalization method was inspected using both smoothed density plots of normalized Ct values and boxplots of Ct relative log expressions. After normalization, a further quality check was introduced by using a correlation matrix to assess inter‐sample Ct correlations using plotCtPairs function. miRNA differential expression was tested using linear modelling with the limma package. After model and contrast fitting, *t*‐statistics were moderated using eBayes function then *p* values and fold changes were extracted using topTable.

### 2.7 Small RNA sequencing microglia

Library preparation and high‐throughput sequencing of retinal microglia RNA was performed by the Biomolecular Research Facility (JCSMR, ANU). Libraries were generated via Capture and Amplification by Tailing and Switching (Diagenode) underwent processing to remove indexes, adapters and template‐switching oligonucleotides using the following cutadapt code: cutadapt –trim‐n ‐a GATCGGAAGAGCACACGTCTG ‐a AGAGCACACGTCTG<input.file>|cutadapt ‐u 3 ‐a AAAAAAAAAAACAAAAAAAAAA ‐e 0.16666666666666666 ‐ |cutadapt ‐g GTTCAGAGTTCTACAGTCCGACGATC ‐m 18 ‐o<output.file>‐

Reads were subsequently aligned to the mouse(mm10) genome using bwa aln then annotated based on genomic coordinates of mature miRNAs sourced from miRbase. Counts for each miRNA were generated with featureCounts and normalised with the Trimmed Means of M (TMM) method. Differential expression analysis was conducted using limma:voom method.

### 2.8 Retinal extracellular vesicle isolation

Following degeneration paradigms, retinas from DR and PD mice were collected for EV isolation as previously described (Wooff et al., 2020). Briefly, 20 retinas from 10 mice were pooled and collected in Hanks Buffered Saline Solution (HBSS, Gibco; Thermo Fisher Scientific, MA, USA). Retinas were transferred to 500 μL digestion solution ((HBSS containing 2.5 mg/mL papain (Worthington Biochemical, NJ, USA), 200U DNase I (Roche Diagnostics, NSW, Australia), 5 μg/mL catalase (Sigma‐Aldrich, MO, USA), 10 μg/mL gentamycin (Sigma‐Aldrich, MO, USA) and 5 μg/mL superoxide dismutase (Worthington Biochemical, NJ, USA)) and finely chopped using scissors. Retinas were incubated at 37°C for 8 min, followed by 20 min at 8°C, to allow for the breakdown of the extracellular matrix and EV release. Following digestion, tissue suspensions were neutralized by diluting in 11.5 mL of HBSS and centrifuged at 1000 × *g* for 10 min at 4°C to remove cells and cell debris. The supernatant was transferred to 14 × 89 mm Beckman Ultra‐Clear ultracentrifuge tubes (Beckman Coulter, CA, USA) and centrifuged at 10,000 × *g* for 30 min at 4°C in a Beckman Coulter Optima XE‐100 (fitted with a SW41Ti Rotor (Beckman Coulter, CA, USA)), to collect large EVs and remaining cell debris. The EV‐containing supernatant was transferred to new ultracentrifuge tubes and centrifuged for 1.5 h at 150,000 × *g* at 4°C. The supernatant was carefully decanted, and the EV pellet was washed in 500 μL Ultrapure Endotoxin‐free 0.1 M PBS before being centrifuged again for 1.5 h at 150,000 × *g* at 4°C. EV populations within this manuscript reflects small‐to‐medium EV (<200 nm) in keeping with MISEV guidelines (Théry et al., 2018), however will be referred to as EV in the manuscript for simplicity. EV were isolated from retinal digestion preparations with >90% viability, which may contribute a small percentage of small apoptotic bodies in the final pellet.

### 2.9 Extracellular vesicle characterization

#### 2.9.1 Transmission electron microscopy

Retinal EV suspensions from DR and PD mice (3 μL) were placed on a 200‐mesh carbon‐coated EV grid (Sigma‐Aldrich, MO, USA) pre‐treated with glow discharge using an PELCO easiGlow™ Glow Discharge Cleaning System as previously described (Wooff et al., 2020). Retinal EV were contrasted with 2% uranyl acetate solution for 1 min, followed by which the grid was blotted to remove excess uranyl acetate and then air‐dried for 5 min. A JEOL JEM‐F200 microscope was used to image EV at 200 kV voltage and 10,000–20,000× magnification. A total of 20 images were captured across four different grids. The images were imported into ImageJ V2.0 software (National Institutes of Health, Bethesda, MD, USA), scale‐calibrated and the diameter of EV measured. The size distribution was plotted in a histogram with 50 nm wide bins using Prism V7.0 (GraphPad Software, CA, USA).

#### 2.9.2 Retinal extracellular vesicle protein isolation

To isolate retinal EV proteins, the resultant EV pellet following ultracentrifugation was resuspended via trituration for 1 min in Pierce® RIPA lysis and extraction buffer with protease cocktail inhibitor (Thermo Fisher Scientific, MA, USA) and frozen at −80C until further use.

### 2.10 Quantitative proteomics

Retinal EV protein was sent to Australian Proteome Analysis Facility (APAF), Macquarie University, NSW, Australia for 1D LC‐MS/MS Tandem Mass Spectrometry using Q‐Exactive HF‐X (Thermo Fisher Scientific) and NanoLC UltiMate 3000 (Thermo Fisher Scientific) systems.

#### 2.10.1 Sample processing

##### Sample digestion

Sample solutions were dried through a brief vacuum centrifugation. Samples were further processed for mass spectrometric analysis using commercially procured S‐Traps (Protifi, USA), as per manufacturer's instructions. Briefly, samples were suspended in S‐Trap lysis buffer (10% SDS, 100 mM triethylammonium bicarbonate, TEAB, pH 8.5). Cysteine disulphide bonds were reduced with 10 mM DTT at 56°C for 30 min, and then alkylated with 25 mM IAA for 30 min in dark at room temperature. The pH of the samples was adjusted using 12% aqueous phosphoric acid, added at 1:10 for a final concentration of ∼1.2% phosphoric acid and diluted using S‐Trap binding buffer (90% aqueous methanol containing a final concentration of 100 mM TEAB, pH 7.55). S‐Trap binding buffer was added to the acidified lysis buffer and the sample mixture was transferred to a labelled S‐Trap column and centrifuged at 4,000 *g*, after which the flow through was discarded.

The column was washed using S‐Trap binding buffer and proteins retained on the column were digested in the presence of 125 μL trypsin solution (∼20ug total trypsin in 50 mM triethylammonium bicarbonate) for 2 h at 47°C. Following digestion, peptides were eluted off the column after an addition of 50 mM triethylammonium bicarbonate and centrifugation. Remaining peptides were eluted from the column using a sequential centrifugation with addition of 0.2% aqueous formic acid followed by 50% aqueous ACN containing 0.2% formic acid. Peptides were dried by vacuum centrifugation. Peptides were reconstituted in mobile phase A (99.9% water, 0.1% formic acid).

#### 2.10.2 Data acquisition and analysis

##### 2.10.2.1 1D data dependent acquisition (DDA) LC‐MS/MS

Peptides were subjected to LC‐MS/MS analysis. Briefly, peptides were injected onto the peptide trap column (C18 PepMap 100, 5 μm, 100 Å (Thermo Fisher Scientific)) and washed with loading buffer (99.9% water, 0.1% formic acid). The peptide trap was then switched in line with the analytical nano‐LC column (Halo‐C18, 160°C, 2.7 μm, 100 μm × 30 cm). Peptides were eluted from the trap onto the nano‐LC column and peptides were separated with a linear gradient of 3.5% mobile phase B (99.9% acetonitrile, 0.1% formic acid) to 25% mobile over 100 min at a flow rate of 600 nL/min, followed by 85% B for 8 min. The column was then re‐equilibrated using 3.5% mobile phase B. Column Temperature was set to 35°C.

The column eluent was directed into the ionization source of the mass spectrometer operating in positive ion mode. Peptide precursors from 350 to 1850 m/z were scanned at 60k resolution. The 20 most intense ions in the survey scan were fragmented by HCD using a normalized collision energy of 28 with a precursor isolation width of 1.3 m/z. Only precursors with charge state +2 to +5 were subjected to MS/MS analysis. The MS method had a minimum intensity threshold value of 4.3e (Newman & Reichenbach, 1996) ions for MS2 triggering, an AGC target value of 3e3 for MS2. MS/MS scan resolution was set at 30,000 and dynamic exclusion was set to 30 s.

#### 2.10.3 Protein identification and quantification

Sequence database searches were performed using the Proteome Discoverer mass informatics platform (version 2.5, Thermo Scientific), using the search programs Mascot and Sequest. Peak lists derived from LC‐MS/MS were searched against Mus musculus sequences in the Uniprot database (210114_UniPr_Mouse_Revi+Unrevi.fasta, containing 63,724 sequences). The parameters for the data processing for peptide identification and quantification were as follows:

Enzyme Name: Trypsin, Maximum missed cleavages: 2, Precursor mass tolerance: 20 ppm. Fragment mass tolerance: 0.02 Da, dynamic modifications: Oxidation (M), Acetyl (Protein N‐Terminus), Deamidated (N and Q), Gln → pyro‐Glu (N‐term Q) and Glu → pyro‐Glu (N‐term E). Static Modification: Carbamidomethyl (C). FDR and result display filters: Protein, Peptide and PSM FDR < 1%. The raw (label free) quantitative data were used for final statistical analysis.

#### 2.10.4 Proteomics data analysis

Raw protein intensity values were log2 transformed then proteins with >2 missing values in one experimental group were removed from the expression matrix. Missing values for the remaining proteins were imputed by randomly selecting values from a Gaussian distribution were μ = 0.0‐th quantile of expression matrix and σ = median of the standard deviation across proteins detected in >50% of the recoded values. This imputation method was selected because missing values in the expression matrix were skewed towards the left end of the expression density curve suggesting that missing values arise from expression below detection threshold.

The imputation procedure was carried out using the impute R package (method = ‘MinProb’) (Trevor Hastie, 2023). Following imputation, the expression matrix was subjected to variance stabilizing transformation, then the normalized values were used for model fitting and differential expression using limma. T‐statistics produced by lmFit, were moderated with the eBayes then retrieved together with logFC values using topTable. To assess the contribution of retinal cell types to the proteome of retinal EV, deconvolution analysis was performed using support vector regression, a method as described by Newman et al. (2015) and implemented in the granulator R package (Rapid benchmarking of methods, 2023). Gene expression signatures for retinal cell types were derived from single cell RNA sequencing and both proteomic data and gene expression values were bound to the [0,1] interval using the following gene‐wise transformation: X_scaled_ = (X—X_min_)/(X_max_ = X_min_) prior to the estimation of cell type contribution.

### 2.11 Single‐cell RNA sequencing

#### 2.11.1 Retinal single cell preparation

Single cell suspensions from mouse retinas undergoing PD for 1, 3 and 5 days together with DR controls were prepared as published previously (Chu‐Tan et al., 2018; Rutar et al., 2015), and FACS‐sorted for viable cell using 4′, 6‐diamidino‐2‐phenylindole (DAPI, Thermo Fisher, MA, USA) staining (1:10000). Each sample comprised of 2 retinas from a single animal. Post‐sort viability was verified again immediately prior to single cell library preparation using the Countess.3 automated counter and manually with a haemocytometer. Only samples with viability >80% were deemed suitable, and 10 (Newman & Reichenbach, 1996) cells were used as input for scRNAseq libraries at 106 cells/mL density.

#### 2.11.2 Library preparation

Single‐cell RNA sequencing libraries were prepared using the Chromium Single Cell 3′ Reagent Kit (10X Genomics) at the ANU Biomolecular Research Facility. Briefly, cells were encapsulated into Gel Bead‐In‐Emulsions (GEMs) using the Chromium Controller (10X Genomics), followed by reverse transcription and cDNA amplification. Sequencing libraries were constructed, quantified, and sequenced to saturation on an Illumina Novaseq6000 platform.

#### 2.11.3 Quality control

Raw sequencing data were processed using the Cell Ranger with default settings (10X Genomics) to generate gene‐barcode matrices. Droplets with less than 200 detected genes and genes expressed in less than five cells were excluded from the expression matrix.

#### 2.11.4 Normalization and clustering

Expression matrices were merged then normalized and scaled using the SCTransform function in the Seurat R package (version 3.2.1) (Stuart et al., 2019). Dimensionality reduction was performed using principal component analysis (PCA) on the top 3000 highly variable genes identified by the SCTransform function (Stuart et al., 2019). The PCA embeddings were then used for the identification of nearest neighbours and clustering by applying the FindNeighbors and FindClusters functions in Seurat using the following key parameters: k.param = 20, dims = 40 and resolution = 0.8.To visualise the identified cell clusters, the PCA embeddings were further dimensionally reduced using the Seurat wrapper of the python umap‐learn package, generating a 2D Uniform Manifold Approximation and Projection (UMAP) plot. Finally, cell labels were assigned to the Seurat clusters using the scType package and its inbuilt marker databases for retinal cell types.

#### 2.11.5 Differential expression and trajectory interference

Slingshot was employed to identify global lineage structures and principal curves in the UMAP space. The starting cluster was specified for the analysis, and the algorithm was constrained to prevent the formation of lineage breaks (allow.breaks = FALSE) and allowed to stretch the principal curves (stretch = 2). Gene expression changes expression along pseudotime trajectories were tested by fitting generalized additive regression models using the tradeSeq algorithm (Van den Berge et al., 2020). The optimal number of knots required fitting was determined by examining the AIC values across 3–15 knots using the evaluateK function. Differential expression between clusters was conducted by fitting negative binomial generalized linear models (glmQLFit) and then performing quasi likelihood F(glmQLFTest) tests in edgeR. Droplet counts were normalized prior to modelling fitting using TMM method.

#### 2.11.6 Ligand receptor interactions and functional analyses

Ligand‐receptor interaction pairs were retrieved from the CellChat database (Jin et al., 2021). To identify the cellular location of receptors targeted by ligands present in the proteomic dataset, the gene expression signature of major retinal cell type was derived from the reanalysis of publicly available single cell data (Fadl et al., 2020) using the processing pipeline described above. After processing, the top 100 most differentially expressed genes in each cell type were created using FindAllMarkers function in Seurat (Stuart et al., 2019) then used as gene set annotation when performing gene set enrichment analysis using fgsea R package. The metric for gene ranking was either average abundance or logFC and 1000 permutations were used to estimate the *p* values. Gene ontology and pathway analysis performed using enrichR used differentially expressed proteins (*p* < 0.05, |logFC| > 0.5) as input and significant terms were summarised by clustering similarity matrices and generating word clouds using the simplifyEnrichment package.

### 2.12 Data availability statement

The data that support the findings of this study ‘Single cell RNA sequencing of mouse retinal cells from generation model’ are openly available in the Sequencing Read Archive NCBI repository under bioproject reference number PRJNA990691.

Raw datasets from Müller miRNA OpenArray and EV proteomics are available in Tables S1 and S4 respectively.

MiRNA expression profiles of wild‐type and DICER knock‐out Müller cells and the transcriptomic profiles of major retinal cell types were retrieved from GSE135835 and GSE153674, respectively (Gene Expression Omnibus).

Microglia miRNA sequencing dataset available in the Sequencing Read Archive NCBI repository under bioproject reference number PRJNA1018286.

### 2.13 Results

#### 2.13.1 Neuronal‐produced miRNA are communicated to Müller glia in response to degeneration

**FIGURE 1:**
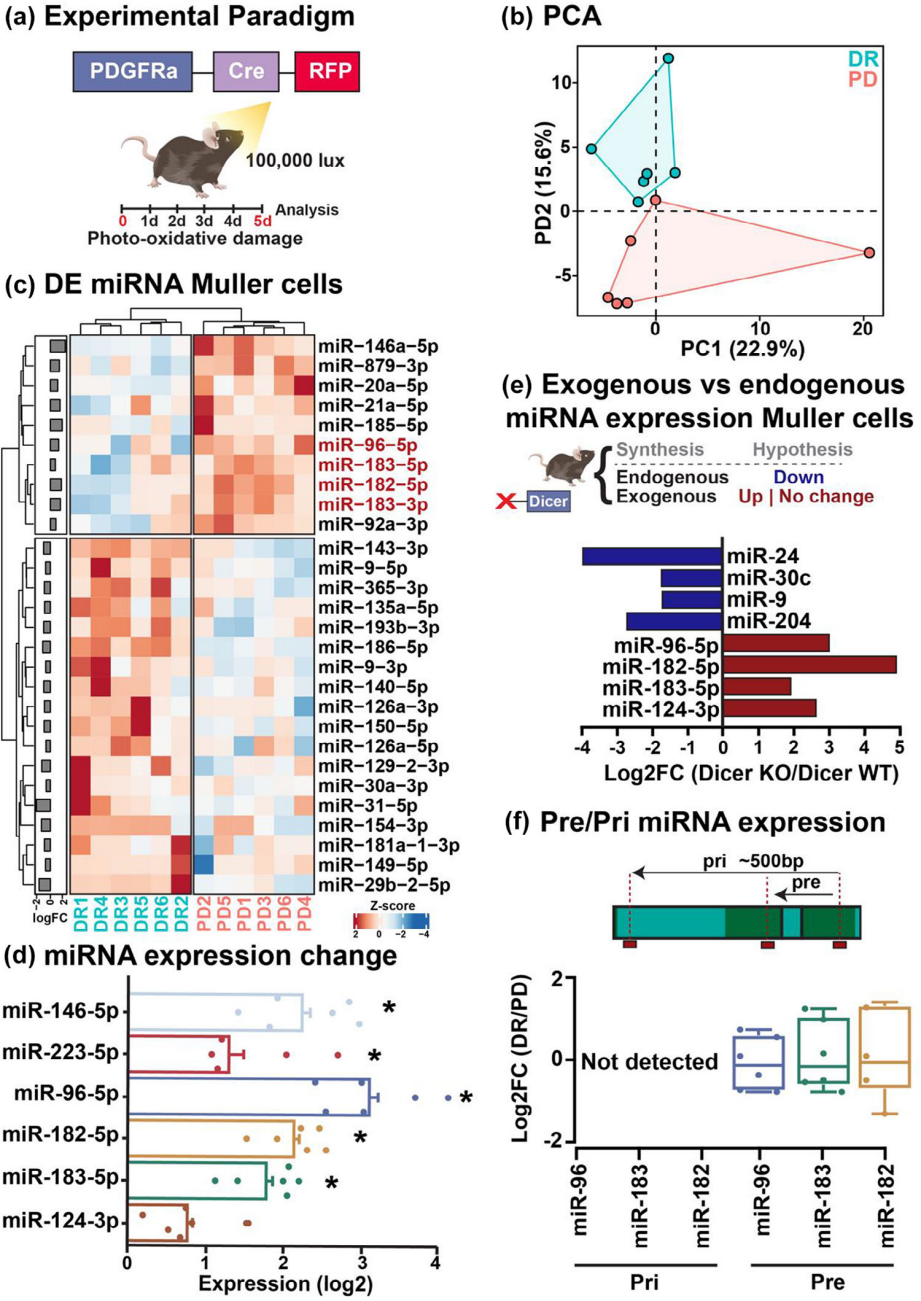
Müller glia miRNA expression and production. (a) Experimental paradigm. PDGFRa‐Cre‐RFP expressing Müller glia were isolated from DR and 5d PD retinas using papain digestion and FACS isolation (*N* = 6). (b) PCA performed on the normalized Ct values of the 139 miRNA detected in in Müller cells. (c) Hierarchical clustering and heatmap from Open Array analysis shows differentially expressed miRNA in Müller glia from 5d PD retinas compared to DR controls (*p* < 0.05, *N* = 6). Bar chat annotation to the left of the heatmap shows the average fold change of each miRNA. Heatmap colours show the *z*‐score of inverted and normalized *Ct* values, with lower values indicating higher expression. (d) qRT‐PCR validation of Müller‐expressing miRNA (*p* < 0.05, *N* = 6). (e) To assess if differentially expressed Müller miRNA were endogenously versus exogenously produced in Müller glia, miRNA was quantified from DICER‐Müller KO mice and compared to WT controls. Downregulation of miRNA in DICER‐ Müller KO mice were hypothesized to be endogenously produced (blue) while an upregulation/no change was hypothesized as an exogenous production (red) (*N* = 6). (f) Validation of exogenous miRNA production was conducted via qRT‐PCR, showing that there was no amplification of pri‐miRNA expression detected in Müller glia, and no change in pre‐miRNA expression for known neuronally produced miRNA miR‐96‐5p, miR‐183‐5p and miR‐182‐5p (*p* > 0.05, *N* = 6).

To unravel miRNA dysregulations associated with gliosis, a miRNA Open‐Array was performed on FACS‐isolated Müller glia from control (dim‐reared; DR) and degeneration (photo‐oxidative damage; PD) conditions in PDGFRa‐Cre‐RFP mice (Figure 1a and Figures S1 and S2a). Standard histological measures were conducted on retinal cryosections to validate the specificity of the strain (Figure S1a) and degenerative features including outer nuclear layer (ONL) thinning, significant photoreceptor cell death and Müller gliosis (Figure S1b‐d, *p* < 0.05). FACS gating strategy and post‐sort populations showed RFP^Hi^ populations expressed Müller markers glutamine synthetase (GLUL/*Glul*) and apolipoprotein E (*Apoe*) (Roesch et al., 2008), (Figure S2a,b). PCA bi‐plot derived from the correlation matrix and computed using the Ct values of the 139 detected miRNA shows clear clustering by DR and PD experimental groups (Figure 1b and Table S1 and S2). From the 139 detected miRNA, 35 were differentially expressed (12 upregulated, 23 downregulated, *p* < 0.05, Figure 1c and Figure S1e), including miR‐146‐5p and miR‐223‐3p, which have previously been reported to regulate inflammation and gliosis (Aggio‐Bruce et al., 2021; Fernando et al., 2020), as well as miR‐183‐5p/3p, miR‐182‐5p and miR‐96‐5p, a photoreceptor‐specific, polycistronic miRNA cluster collectively referred to as miR‐183/96/182 (Krol et al., 2010) (Figure 1c and Figure S3, *p* < 0.05). Validation of upregulated miRNA was verified using qRT‐PCR showing significant upregulation of miR‐146‐5p, miR‐223‐3p and miR‐183/96/182, with no change in miR‐124‐3p, a known miRNA highly expressed in retinal neurons but not specific to photoreceptors (Sun et al., 2015) (Figure 1d, *p* < 0.05). Noting the upregulation of photoreceptor‐specific miR‐183/96/182 in Müller glia, the origin of miR‐183/96/182 production was explored to investigate if these known neuronal miRNA were synthesized, or exogenously transported to Müller glia in response to degeneration. To test if miR‐183/96/182 are endogenously or exogenously transcribed, we explored the miRNA of Müller glia following the conditional deletion of DICER using a dataset previously published by Wohl et al. (2017). As DICER is required for miRNA maturation and cleavage to the active mature form O'Brien et al. (2018), DICER KO Müller glia should not be capable of endogenous miRNA production. Consistent with this rationale, conditional DICER deletion resulted in the upregulation of miR‐183/96/182, and miR‐124‐3p, while glial‐enriched miRNA such as miR‐204, miR‐30, miR‐9 and miR‐24 were robustly downregulated, as reported in the original study (Wohl et al., 2017) (Figure 1e). Finally, to further validate this result, the expression of both pri‐ and pre‐miR‐183/96/182 (primary and precursor forms of miRNA that can only be increased in the producing cell) was compared between DR and PD PDGFRa‐Cre RFP‐isolated Müller glia. No pri‐forms were detected in either DR or PD samples, suggesting a lack of endogenous miRNA transcription and no change in expression was seen in precursor miRNA transcripts between DR and PD groups (Figure 1f, *p* > 0.05). These results combined support an exogenous contribution of mature forms of neuronally‐produced miRNA to Müller glia cells in response to degeneration, potentially as an attempt to mitigate or control gene expression pathways associated with inflammation and gliotic responses.

#### 2.13.2 Neuronal‐produced miRNA are downregulated in microglia in response to degeneration

**FIGURE 2:**
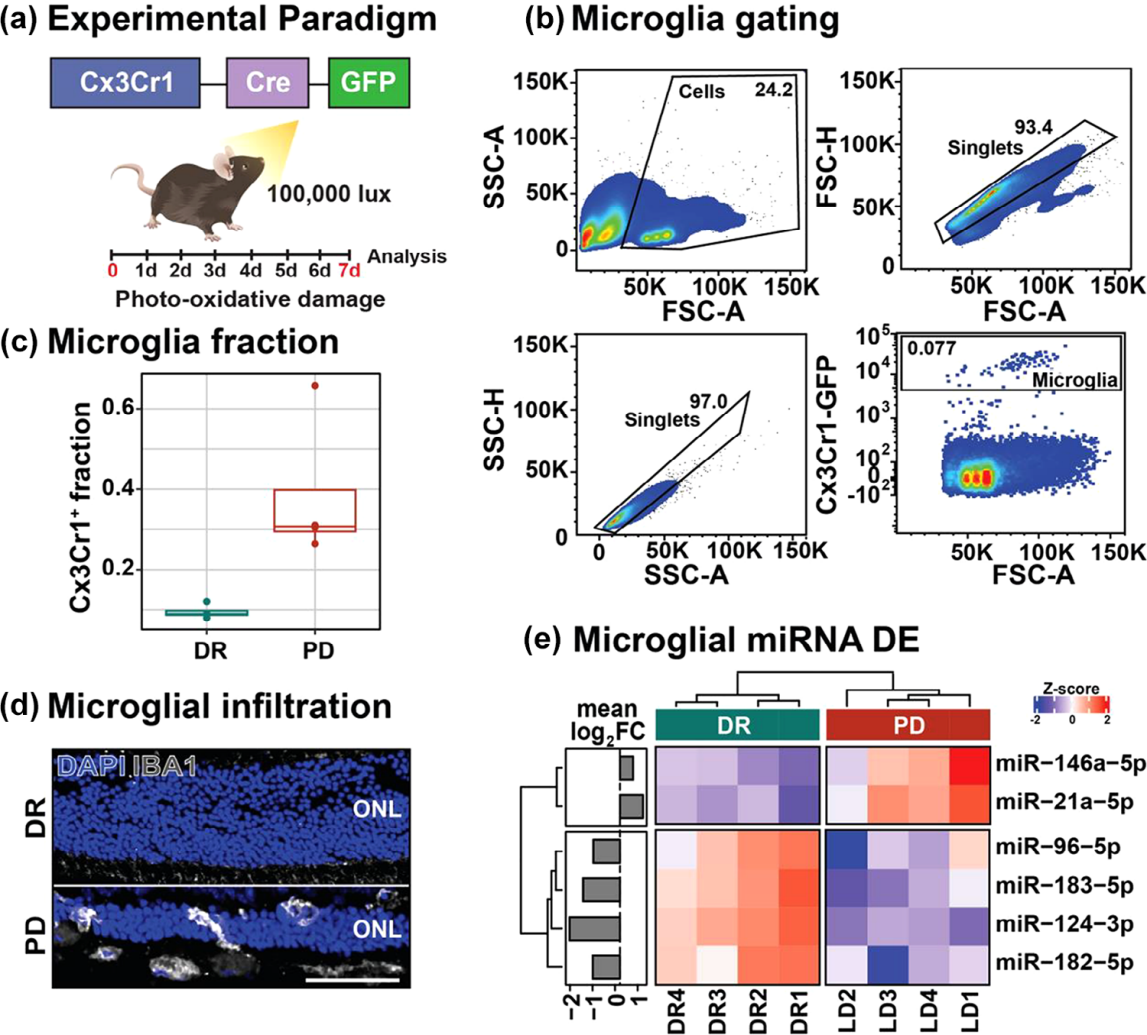
Microglia/macrophage miRNA expression. (a) Experimental paradigm. Cx3Cr1‐Cre‐GFP expressing microglia/macrophage were isolated from DR and 7d PD retinas using papain digestion and FACS isolation (*N* = 4). (b) FACS gating strategy. (c) Cx3Cr1^+^ microglial/macrophage cells from DR and 7d PD retinas shows increased Cx3Cr1^+^ cells following PD (*p* < 0.05, *N* = 4). (d) Increased presence of IBA‐1^+^ microglial/macrophage cells in the outer retina following PD. (e) Differential expression of microglial/macrophage miRNA shows significant downregulation of neuronal miRNA miR‐96‐5p, miR‐183‐5p, miR‐124‐3p and miR‐182‐5p and significant upregulation of glial miRNA miR‐146a‐5p and miR‐21a‐5p. (*p* < 0.05, *N* = 4). ONL, outer nuclear layer. Scale = 50 μM.

The expression of neuronally‐produced miRNA in another retinal glia type, microglia, was next explored by isolating microglia/macrophage populations from DR and 7d PD Cx3Cr1^+^‐Cre GFP mice using FACS and performing small RNA sequencing (*N* = 4, 6—12 retinas per sample), (Figure 2a,b). A significantly increased number of Cx3Cr1^+^ microglia/macrophages were isolated from PD retinas, reflecting the increased inflammatory environment and immune cell recruitment known to occur in this model (Natoli et al., 2016) (Figure 2c,d, *p* < 0.05). Differential expression analysis of miRNA between DR and 7d PD groups showed that while there was a significant increase in expression of known microglial/macrophage miRNA, miR‐146a‐5p and miR‐21a‐5p; neuronal miRNA including miR‐124‐3p and the photoreceptor cluster miR‐183/96/182, were significantly decreased in microglial/macrophage cells in 7d PD samples (Figure 2e, *p* < 0.05). Overall, this result suggests that neuronal miRNA may either not be targeted to microglial/macrophage cells or have decreased communication to these cells in response to degeneration.

#### 2.13.3 Neuronal miRNA enriched in retinal EV modulate gliotic inflammatory responses

**FIGURE 3:**
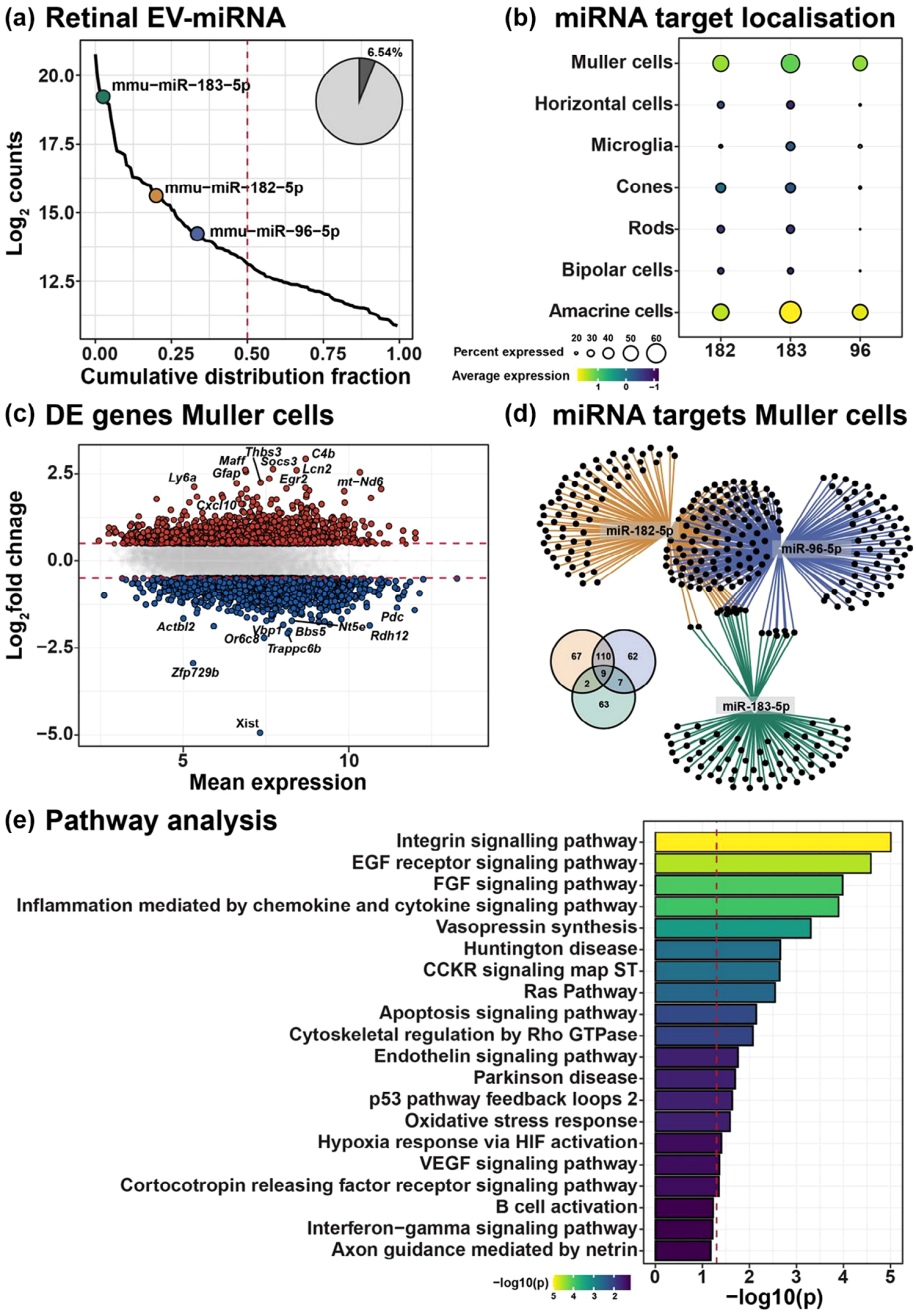
Neuronal miRNA enriched in retinal EV modulate Müller gliosis. (a) Photoreceptor‐produced miRNA miR‐183/96/182 were significantly enriched in retinal EV (red‐dashed line representing top 50% of all EV‐miRNA). (b) mRNA targets of miR‐183/96/182 were found to localize primarily to Müller glia, and amacrine cells in the retina. MiRNA targets retrieved from TargetScan, and retinal cell transcriptome were generated by reanalysis of a single cell RNA sequencing data reported by Fadl et al. (2020). (c) Volcano plot of differentially expressed genes in Müller glia, from DR and PD retinas using a publicly available dataset (Wohl et al., 2017), (significance cut off FC > 0.5). (d) Network analysis and Venn diagram of miR‐183/96/182 differentially expressed mRNA targets. (e) Pathway analysis of miR‐183/96/182 targets highlight the association with biological processes that define gliosis including cytoskeletal changes, inflammation, oxidative stress, apoptosis, neurodegeneration, growth factor signalling, hypoxia and angiogenesis.

To further explore the mechanism by which exogenous transfer of photoreceptor/neuronally produced miRNA are transported to glia cells in response to degeneration, the role of extracellular vesicle (EV)‐mediated transport was investigated as the primary mechanism facilitating increased expression of miR‐183/96/182 in retinal Müller glia. EV have known roles in the selective incorporation and intracellular delivery of molecular cargo including miRNA (Théry et al., 2018). Previous results from our lab have in fact shown reduced transport of neuronally‐produced miRNA, miR‐124‐3p, to Müller glia, in EV‐inhibited mice (Wooff et al., 2020). To validate whether EV‐miRNA cargo contained miR‐183/96/182, we probed our previous dataset profiling miRNA content in EV isolated from healthy retinal tissue and showed that miR‐183/96/182 were found amongst the top 30% most highly enriched miRNA in retinal EV, together accounting for 6.54% of the total EV‐miRNA cargo (Figure 3a). Next, we explored the regulatory power of miR‐183/96/182 across retinal cell types by examining the expression of their targets (retrieved from TargetScan) and data from a recent single cell sequencing study defining the transcriptome of major retinal cell types in healthy C57BL/6J mice (Fadl et al., 2020). Gene expression modules comprising targets of miR‐183/96/182 were found to be most highly enriched in Müller glia and amacrine cells, followed by photoreceptor cones and rod cells as well as microglia and other retinal interneurons (Figure 3b, *p* < 0.05). To further define the regulatory roles of miR‐183/96/182 in Müller glia, we intersected the targetome of these miRNA with differentially expressed genes (|logFC| > 0.5) in gliotic Müller cells purified from control and PD retinas reported in work by Kang et al. (2021). The presence of gliosis is confirmed by both the strong *Gfap* upregulation, a characteristic marker of gliosis, as well as several proinflammatory mediators including *Cxcl10*, *Socs3* and *C4b* (Figure 3c). Network analysis performed on differentially expressed targets of miR‐183/96/182 identified both unique as well as shared targets between the miRNA (Figure 3d and Table S3). Pathway analysis on the combined network points to the involvement of miR‐183/96/182 targets in biological processes that define gliosis including ‘regulation of inflammation via cytokine/interferon signalling’, ‘aberrant growth factor (EGF and FGF) signalling’, ‘cytoskeletal changes mediated by Rho signalling’, ‘establishment of a hypoxic (HIF‐signalling) and pro‐angiogenic environment (VEGF‐signalling)’. In addition, biological pathways associated with neurodegeneration (Parkinson's and Huntington's disease), apoptosis and integrin signalling were noted, the latter having key roles in EV uptake and tropism. Taken together, the integration of EV‐miRNA data with the transcriptome of gliotic Müller cells highlights the enrichment of miR‐183/96/182 in retinal EV and the potential capacity of these miRNA to shape the gliotic response in other models of degeneration with similar pathological features.

#### 2.13.4 Retinal EV proteome is differentially expressed in response to photo‐oxidative damage‐induced degeneration

**FIGURE 4:**
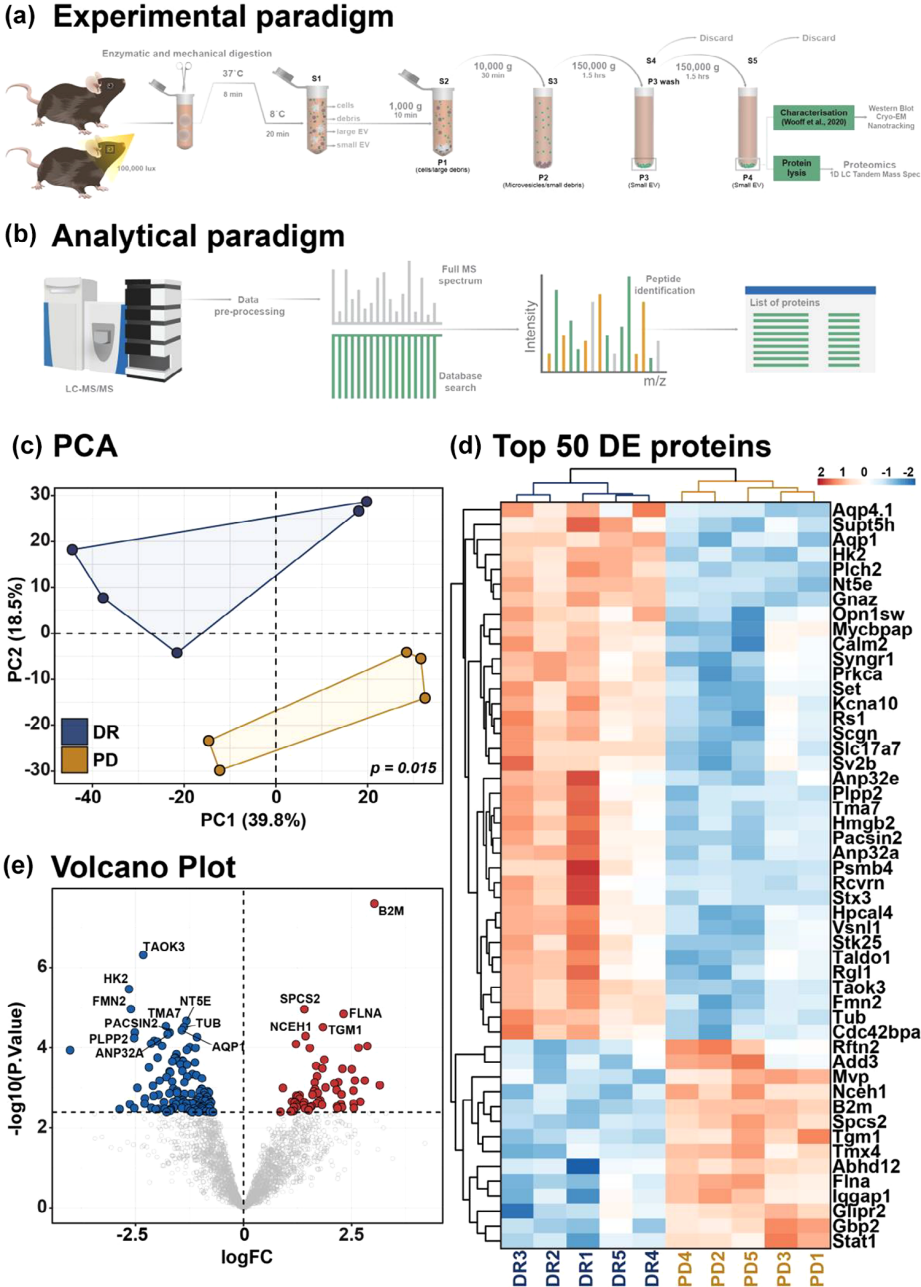
Differentially expressed retinal EV proteome following photo‐oxidative damage‐induced retinal degeneration. (a) Experimental paradigm to isolate retinal EV from DR and PD mice. (b) Analytical paradigm for 1D LC‐MS/MS and protein identification. (c) PCA performed on the shows clear clustering of DR and PD EV samples. (d) Heat map and hierarchical clustering of top 50 differentially expressed retinal EV proteins. (e) Volcano plot shows 119 downregulated and 61 upregulated retinal EV proteins. (*N* = 5, *p* < 0.05).

**FIGURE 5:**
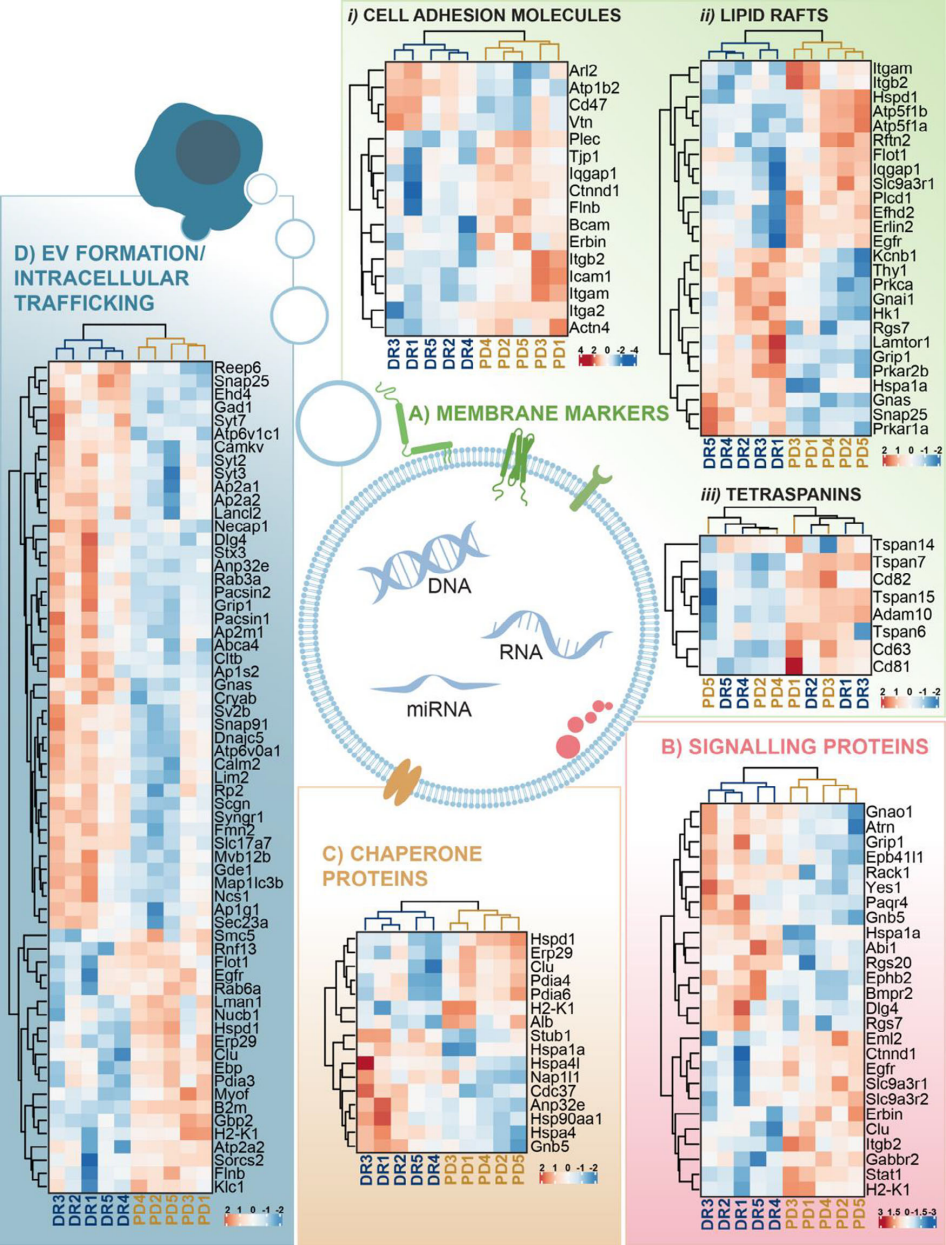
EV signalling, and transport are altered in response to photo‐oxidative damage‐induced retinal degeneration. (a‐d) Heat maps and hierarchical clustering of (a) EV membrane markers, (b) signalling proteins, (c) chaperone proteins and (d) EV formation and intracellular trafficking, collectively show altered EV signalling responses to degeneration. (*N* = 5, *p* < 0.05).

We have shown three key results from this study that support a role for EV‐mediated neural‐to‐glial crosstalk and miRNA transfer in response to degeneration. (1) Retinal EV were found to be enriched with photoreceptor miRNA including miR‐183/96/182, (2) miR‐183/96/182 were trafficked to Müller glia via EV in degeneration and (3) these miRNA were associated with the regulation of gene expression programs associated with gliosis. However, what is currently unclear is how EV signalling may be changed in degeneration to allow for enhanced tropism of EV towards Müller glia. Since the proteomic decorations of EV mediate tropism, we isolated EV from the DR and PD retina and applied LC‐MS/MS to explore their proteomic signature (Figure 4a,b, Figure S4 and Table S4 and S5). Retinal EV were characterized using transmission electron microscopy (TEM), Nanotracking analysis, and identification of classical EV markers (Théry et al., 2018) (Figure S5). PCA bi‐plot derived using the expression values of all identified proteins showed clear clustering by experimental condition (Figure 4c), with the top 50 differentially expressed proteins shown by heat map (Figure 4d, *p* < 0.05), and volcano plot (Figure 4e, *p* < 0.05). In total, 180 proteins were found to be differentially expressed, with 61 upregulated and 119 downregulated (Table S6). Pathway analyses showed that EV proteins which were downregulated were involved in homeostatic retinal processes including phototransduction, visual perception, membrane organization and mRNA stability, while EV proteins which were upregulated were associated with inflammatory pathways and metabolism, including immune responses, interferon and NF‐κB signalling, proton transport and apoptotic signalling (Figure S6a,b). Finally, to investigate if EV communication pathways were altered in degeneration, EV membrane markers proteins (Figure 5a), as well as proteins involved in EV signalling and binding (Figure 5a‐c), and formation and trafficking (Figure 5d) were compared between DR and PD groups. Results demonstrated that while there was no significant difference in the expression of tetraspanin proteins between groups, there was a clear alteration in the expression of cell adhesion molecules, lipid rafts, intracellular signalling, chaperone and EV formation and trafficking proteins in PD samples compared to DR controls (Figure 5a‐d). Collectively these results suggest that retinal EV proteins are required under healthy conditions to regulate homeostatic processes required for vision, but in degeneration mediators of EV formation, signalling, adhesion and transport undergo expressional changes that potentially alter EV tropism.

#### 2.13.5 Selective enrichment of glial marker expression in EV in response to photo‐oxidative damage‐induced degeneration

**FIGURE 6:**
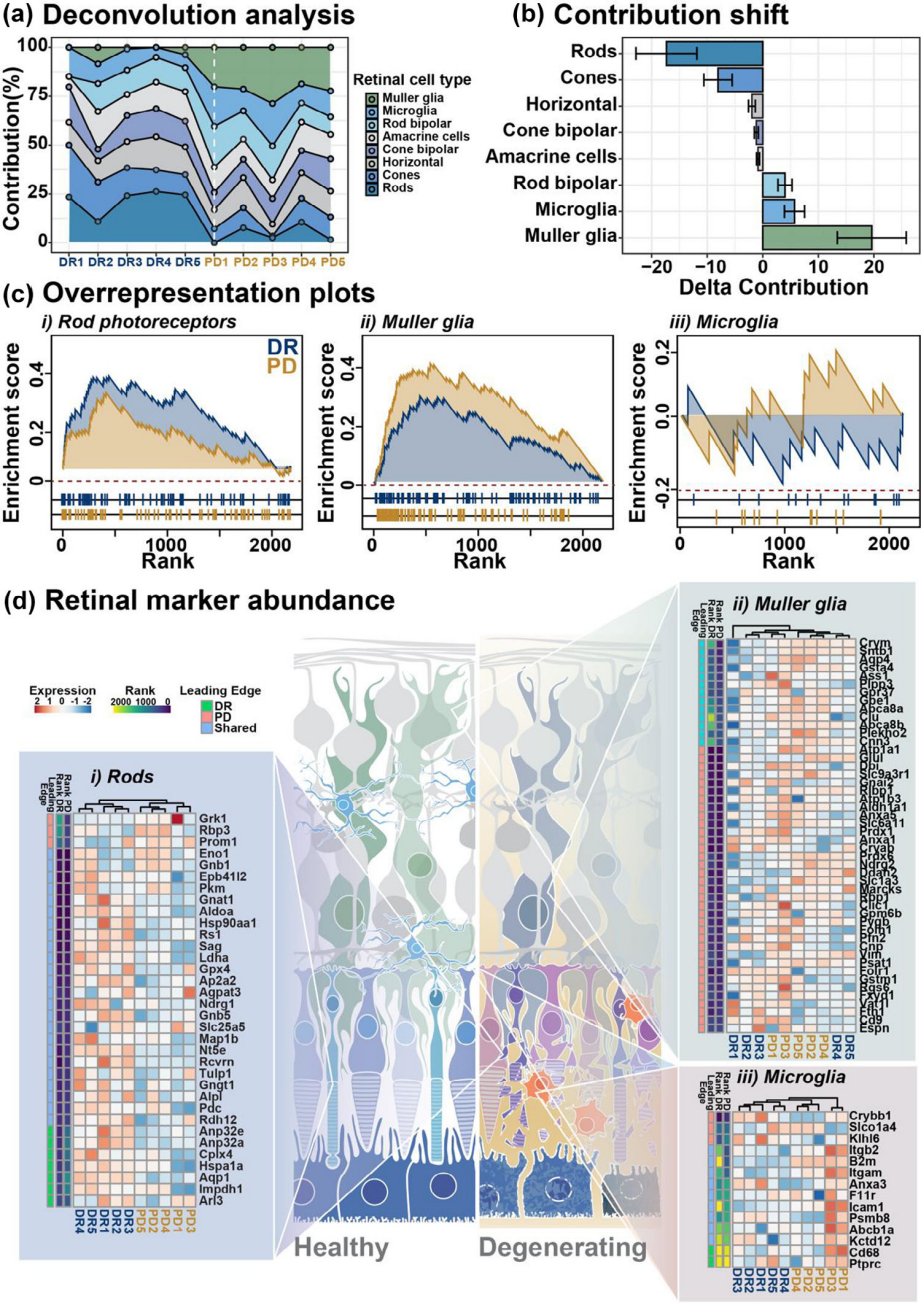
Selective enrichment of glial marker expression in EV in response to photo‐oxidative damage‐induced degeneration. (a, b) Deconvolution and comparative shift analyses for retinal cell type‐specific markers in retinal EV shows a reduction in neuronal markers and enrichment of glial cell markers, specifically Müller and microglia, in EV from PD mice compared to DR controls. (c) Gene set enrichment plots show cell‐type specific protein expression in (*i*) rod photoreceptors, (*ii*) Müller glia and (*iii*) microglia in DR and PD conditions; further highlighting a shift to glial marker enrichment in PD. (d) Heat maps and hierarchical clustering of proteins differentially expressed in (*i*) rods, (*ii*) Müller glia and (*iii*) microglia. Annotations to the left of the heatmaps show the rank of each protein in the GSEA analysis, and whether each protein is associated with the leading‐edge gene subset in DR or PD samples. The leading‐edge represents the genes that occur in the ranked list either at, or prior to, the point where the running sum (shown in c) reaches the highest deviation from zero.

Having demonstrated that PD induces large scale proteomic changes in particular in EV signalling, we investigated whether the loss of photoreceptor cells and/or the inflammatory activation of retinal glia induced by PD could account for the changed EV proteomic cargo and altered signalling responses. Since EV express markers of the host cells from which they are produced (Wohl et al., 2017), we applied deconvolution analysis to approximate the individual contributions of key retinal cell type to the proteome of EV and discern whether alterations in the EV proteome could be ascribed to a particular retinal cell type. The cell type specific gene expression information (Figure S7 and Table S7) required in the deconvolution analysis was derived from the reanalysis of a publicly available single cell RNA sequencing data set published by Fadl et al. (2020), reporting on the transcriptomics of the heathy C57Bl/6J mouse retinal cells (Fadl et al., 2020). Deconvolving the proteomic cargo of EV revealed that in the DR retina the retinal neuronal cells including from photoreceptors, as well as bipolar cells, were the predominant contributors of EV accounting for 38.3% and 23%, respectively. Conversely, in PD EV, the contribution estimates of photoreceptors decreased to 12.9% while Müller and microglial markers increased from 2.6% to 22.2% and 9.5% to 15.2%, respectively (Figure 6a and Table S8, *p* < 0.05). Comparative contribution analysis showed a shift from neuronal to glial markers expression in EV following degeneration (Figure 6b, *p* < 0.05). To identify specific proteins associated with the contribution of photoreceptors, Müller glia and microglia overrepresentation analysis was conducted using a custom‐made reference gene set comprising marker genes (identified by differential expression) for these cell types (Table S7). Consistent with the deconvolution analysis, the over‐representation data further demonstrate enrichment for glial markers in PD and a reduction in the enrichment score associated with photoreceptor markers (Figure 6c). Retinal protein abundance shift in EV is further demonstrated in heatmaps (Figure 6d). These results together suggest a shift to glial‐produced EV as a response to degeneration, either through a comparative decrease in photoreceptor‐derived EV as previously hypothesised (Wooff et al., 2020), an increase in glial‐derived EV populations, or more likely, both.

#### 2.13.6 Predicted ligand‐receptor interactions support glial targeting of retinal EV in response to photo‐oxidative damage‐induced degeneration

**FIGURE 7:**
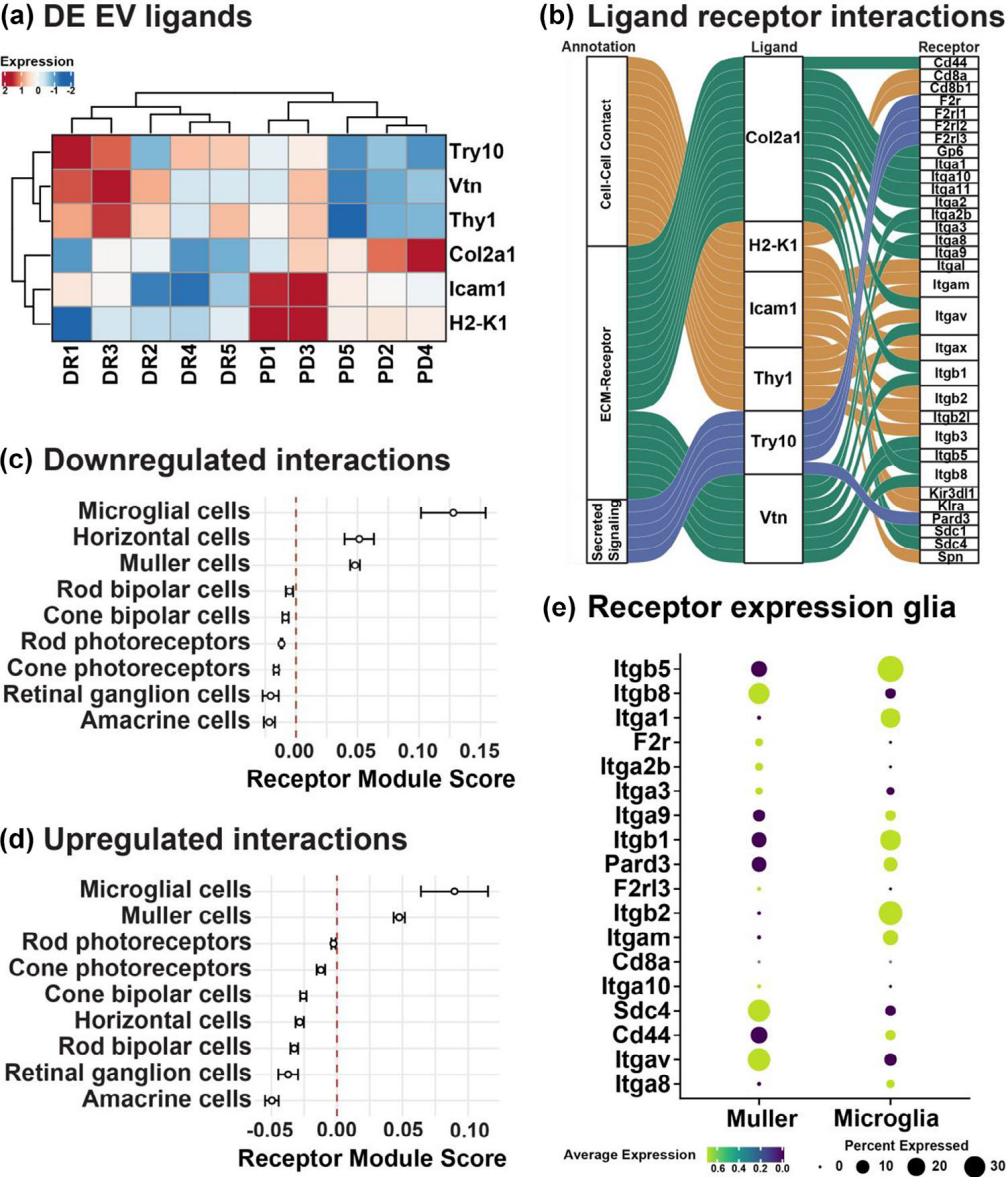
Glial targeting of retinal EV in response to photo‐oxidative damage‐induced degeneration. (a) Heat map showing differentially expressed proteins annotated as ligands in the CellChat database. (b) Predicted receptor interactions of differentially expressed EV ligands based on the receptor‐ligand pairs listed in the CellChat database. (c, d) enrichment scores for receptor modules comprising the receptors (from b) of (c) downregulated ligands and (d) upregulated ligands across retinal cell types showing preferential expression of these receptors in retinal glial cells. (e) Expression of each receptor (from b) in retinal Müller glia and microglia. (*N* = 5, *p* < 0.05).

Having postulated that EV‐dependent miRNA trafficking to retinal glia is altered in response to degeneration, we sought to identify specific mediators of EV tropism. As EV uptake can occur through specific surface ligand‐cell receptor binding mechanisms, we identified such receptor ligand interactions by simultaneously probing the EV proteome for ligands and the retinal cell‐specific transcriptome for their respective receptors. Evidence for ligand‐receptor binding partners was sourced from CellChat, a manually curated receptor ligand interaction database containing ∼2000 interactions (Jin et al., 2021). Limiting the search to differentially expressed EV proteins, we found six ligands that were differentially expressed in EV (Figure 7a). These ligands, TRY10, VTN, THY1 (downregulated) and COL2A1, ICAM1, H2‐K1 (upregulated) had collectively 32 potential receptors, predominantly belonging to the integrin family of adhesion mediators, and formed 40 possible interactions (Figure 7b). Next, we created two gene expression modules by separating the receptors for upregulated and downregulated ligands and assessed the expression of these modules across retinal cell types using single cell sequencing data (Fadl et al., 2020). Both modules showed positive associations with retinal Müller glia and microglia and negative associations with retinal neurons (Figure 7c,d, *p* < 0.05). Finally, while these cell types shared the expression of some receptors, many showed selective enrichment in one glial type indicating some preference for EV targeting to one glial cell over the other (Figure 7e, *p* < 0.05). Integrating proteomic and transcriptomic data from EV and major retinal cell types suggests that photo‐oxidative damage alters the expression of EV ligands that target glial‐enriched receptors which may represent a potential mechanism by which neuronal‐to‐glial molecular cargo exchange occurs in degeneration.

#### 2.13.7 Temporal expression changes in EV signalling pathways in retinal neurons indicate attempts to maintain communication during degeneration

**FIGURE 8:**
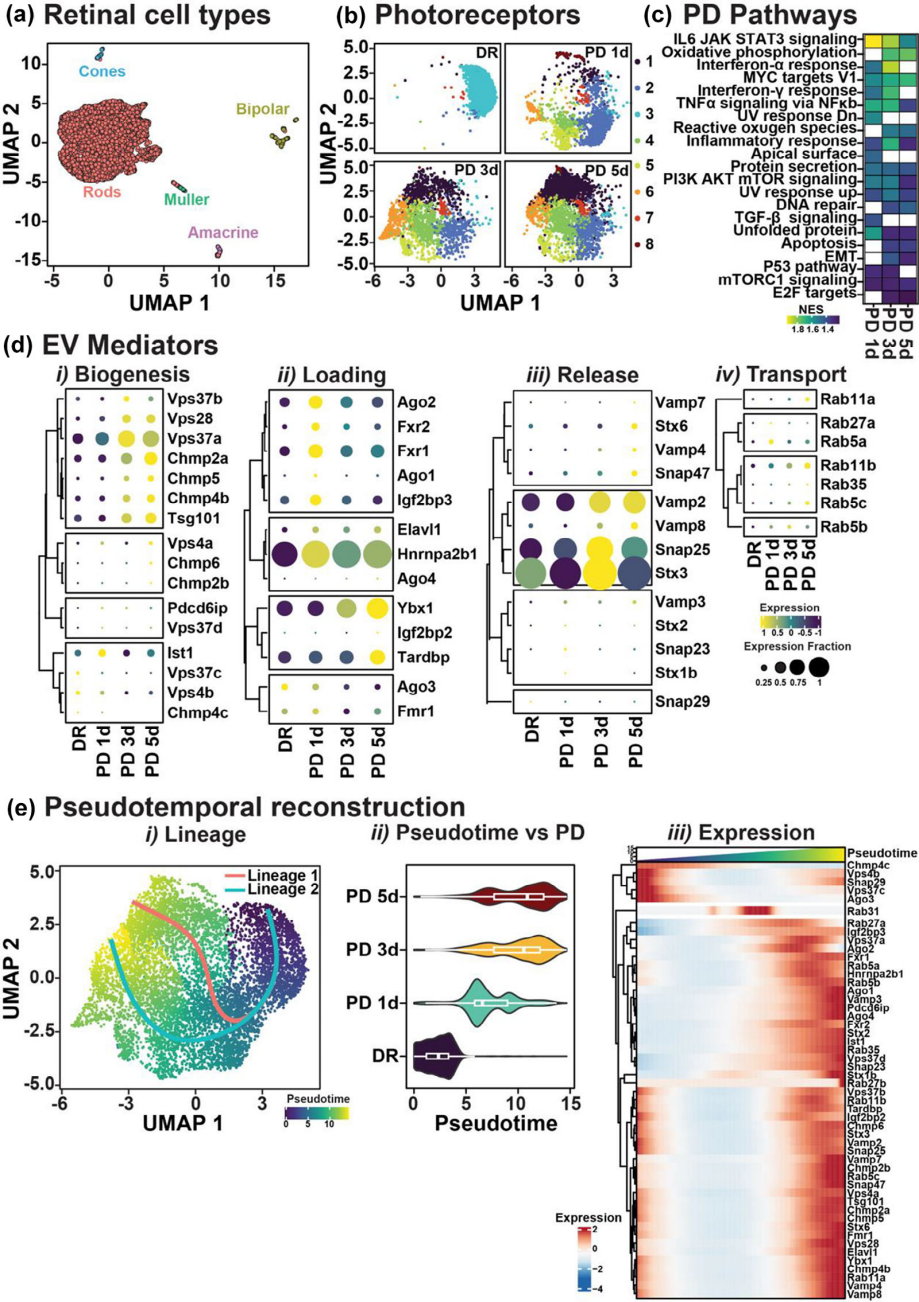
Temporal expression changes in EV biogenesis, loading and release pathways in retinal photoreceptors across PD. (a) UMAP showing major retinal cell types. (b) UMAP showing photoreceptor homogeneity in DR retinal samples and the segregation of photoreceptors into multiple clusters following PD. (c) Pathway analysis of photoreceptor genes across PD broadly indicating the presence of oxidative stress, inflammation and DNA damage and apoptotic pathways. (d) Temporal changes in EV mediators across PD show progressive increase in *(i)* EV biogenesis across PD compared to DR, *(ii)* increased loading from 1d PD, *(iii)* increased EV release reaching maximum expression at 3d PD and *(iv)* increased EV transport progressively across PD. (e) *(i)* Pseudotime interference analysis showing the gradual transition of photoreceptors from a heathy state (DR) to degenerative (PD) along two distinct lineages*. (ii)* Distribution of pseudotime values for photoreceptors from DR and PD retinas, showing a rapid increase between DR and 1d PD, and 1d PD and 3d PD but no further changes between 3d and 5d PD. *(iii)* The relationship between pseudotime changes and gene expression for mediators of EV biogenesis, loading release and transport.

Finally, results from this study have provided evidence that photoreceptor/neuronal degeneration is associated with changes in EV proteomic cargo, bioavailability and potential uptake and tropism to glial cells. Therefore, we explored whether these changes in molecular composition result from dysregulation of EV biogenesis, transport, loading or release processes in photoreceptors as a consequence of degeneration. To gain insight into the gene expression landscape of photoreceptors in retinal degeneration, we performed single cell RNA sequencing on retinas following 1, 3 and 5 days of PD (Figure 8a,b, Figure S8 and Table S9). Pathways analysis on differentially expressed genes at 1‐, 3‐ and 5‐days PD compared to DR controls highlight key features of photoreceptor stress including apoptosis, proinflammatory changes, metabolic dysregulations, and the presence of oxidative damage (Figure 8c). Concurrently, we selected genes involved in EV signalling and cargo loading pathways and verified their roles using STRING (Szklarczyk et al., 2015) pathway and network analysis (Figure S9 and Table S10). We showed that mediators of EV cargo loading were most highly expressed at 1d PD, and EV release mediators were upregulated from 3d PD. EV biogenesis and transport genes were also shown to increase progressively across degeneration in comparison to DR controls (Figure 8d, *p* < 0.05). These results support an increase in EV cargo loading early on in degeneration, with EV release occurring at 3d PD, which we have previously shown in this model to reflect the peak of photoreceptor cell death and inflammation (Wooff et al., 2023). Last, we applied trajectory interference analysis to understand the temporal characteristics of the gene expression changes associated with the dysregulation of EV biology in photoreceptors. Two trajectories were identified describing the transition from health to degeneration in photoreceptors and highlighted a marked increase in pseudotime values between control and 3d PD, with no further pseudotime progression noted at 5 days (Figure 8e and Table S11). Fitting a generalized additive model for the expression of EV biology mediators as a function of pseudotime indicated a simultaneous and rapid upregulation of these genes at pseudotime values describing the transition from 3d PD to 5d PD, with no indication of regression towards baseline over the pseudotime range available (Figure 8e). These results support increased loading of EV cargo as an early response to degeneration with EV release and transport occurring primarily in the late‐phase of degeneration. In summary, results in this work using multiomic integration demonstrate a novel role for EV in mediating neuronal‐to‐glial communication networks, with the transfer of selectively encapsulated neuronal miRNA cargo required for the modulation of homeostatic and gliotic states.

### 2.14 Discussion

Gliosis plays a key role in the propagation of inflammation, and in the progression of retinal and neurodegenerations. Understanding how gliotic responses are initiated and regulated is therefore vital to allow for the identification of therapeutic targets to dampen aberrant inflammatory cascades and prevent disease progression. Building upon our previous findings (Chu‐Tan et al., 2021, 2018; Wooff et al., 2020, 2023), results from this study provide strong evidence to support an EV‐mediated communication axis between neurons and glia, and reveal alterations in EV signalling and EV‐miRNA cargo loading to preferentially target Müller glia in degeneration. Specifically, our results in the retina demonstrate that neuronally‐produced miRNA from photoreceptors may be transported to Müller glia to exert regulatory functions in modulating gliotic responses during degeneration. This work provides evidence for key molecular dynamics governing photoreceptor‐Müller glia EV‐dependent communication—a vital prerequisite for the development of cell specific therapeutics targeting glia applicable to both retinal and neurodegenerations.

#### 2.14.1 Neuronal‐to‐glial communication axis is mediated by EV

Comparable to astrocytes in the brain (Newman & Reichenbach, 1996; Siracusa et al., 2019), Müller glia are the principal macroglia of the retina and display a radial morphology ensheathing all neurons as well as blood vessels as they span the entire length of the neural retina (Newman et al., 2012; Reichenbach & Bringmann, 2013). It is through this network of processes that Müller glia function to provide both architectural as well as homeostatic support to the retina and inflammatory responses (Bringmann et al., 2006). Under conditions of stress or disease, glial cells including Müller glia, respond by undergoing gliosis—a process defined by rapid cytoskeletal changes, the release of inflammatory cytokines and chemokines, and a loss of homeostatic functioning, culminating in neuronal cell death (Eastlake et al., 2016; Kauppinen et al., 2012; Kumar et al., 2013; Olivares‐González et al., 2021; Rübsam et al., 2018; Rutar et al., 2015). Despite an understanding of the triggers, physiological changes, and consequences of gliosis; the control of this process across the CNS and in particular the coordination of inflammatory responses, have remained largely unknown. New insights from this work support a mechanism of EV‐mediated neural‐to‐glial communication underpinning glial inflammatory regulation, with dysregulation in this communication axis leading to pathological gliotic responses known to contribute to photoreceptor cell death.

We demonstrated in this work that under normal healthy conditions in the retina, EV proteins were associated with pathways maintaining homeostasis including phototransduction and visual perception. However, following degeneration, EV signalling pathways were differentially regulated, likely favouring selective targeting to Müller glia, and were associated with pathways involved in immune modulation and apoptotic signalling. We also noted a shift in the composition of EV following degeneration, with a reduction in photoreceptor markers and enrichment in Müller and microglial cell markers in EV, reflecting that photo‐oxidative damage may predominantly alter the photoreceptor and glial EV composition and/or number. As our previous work has demonstrated that photoreceptors may contribute a significant proportion of EV out of the total retinal EV pool, with progressive loss of EV bioavailability positively correlated to photoreceptor cell death (Wooff et al., 2020), these results collectively suggest either a reduction in the numbers of photoreceptor‐derived EV, or a loss of photoreceptor‐EV along with an upregulation of Müller and microglial‐EV populations. Similar findings were also reported in Alzheimer's disease, with significantly enriched glial marker proteins and reduced neuronal proteins found in EV compared to control patients (Muraoka et al., 2020). Based on these findings we propose a mechanism by which neuronal/photoreceptor‐EV are communicated throughout the retina under normal healthy conditions to regulate homeostasis and immune modulation, but in degeneration to compensate for increased inflammatory activation, are preferentially targeted to glial cells to transfer required molecular cargo including potent gene regulator miRNA, to aid in responding to aberrant inflammatory pathway responses. However, when EV communication and the transfer of regulatory molecular cargo can no longer be adequately maintained, pathological levels of inflammation set in, as demonstrated by our previous findings with EV inhibition in vivo leading to exacerbated levels of glial‐mediated inflammation and photoreceptor cell death (Wooff et al., 2020).

We further speculate that increased production of glial‐derived EV may occur as a physiological response to inflammatory stimuli, which has been well characterised to occur in inflammatory cells in the CNS following stress (Frühbeis et al., 2013; Kumar et al., 2017; Verderio et al., 2012; Yang et al., 2018), and further propagate inflammatory cascades (Delpech et al., 2019; Spiers et al., 2022). Dysregulation of EV communication has been linked to the development and progression of neurodegenerations, including Parkinson's and Alzheimer's diseases, where EV are implicated in the transfer of pathological cargo such as α‐synuclein, tau protein, and amyloid‐beta (Gassama & Favereaux, 2021; Muraoka et al., 2020; Spiers et al., 2022). In support of this hypothesis, inhibition of EV has been shown to attenuate inflammatory propagation in the brain, with A‐SMase knock‐out mice shown to be protected against the development of multiple sclerosis‐induced by autoimmune encephalomyelitis (EAE), attributed to reduced inflammatory signal propagation from microglia/macrophages‐derived EV (Verderio et al., 2012). Additionally, in vivo inhibition of EV biogenesis was also linked to reduced amyloid plaque formation, and reduced tau propagation in mouse models of Alzheimer's disease (Delpech et al., 2019). Our results support this hypothesis, demonstrating that along with enriched populations of Müller and microglia glial‐derived EV in degeneration, differentially upregulated EV proteins were associated with inflammatory pathway modulation and cell death.

These findings collectively highlight the role of EV in healthy and pathological states suggesting that restoring regulatory EV communication pathways and cargo is an ideal therapeutic strategy to reduce inflammation and slow the progression of retinal and neurodegenerations.

#### 2.14.2 Selective neuronal EV‐miRNA cargo regulates gliotic inflammatory responses in degeneration

Dysregulation of EV‐miRNA communication has been shown in various neuroinflammatory conditions (Marostica et al., 2021), occurring in response to traumatic injury (Jiang et al., 2020), inflammatory neurodegeneration (Men et al., 2019; Prada et al., 2018; Veremeyko et al., 2019), neuroexcitatory toxicity (Men et al., 2019), alterations in vascular integrity (Xu et al., 2017) and in retinal degenerations (Wooff et al., 2020). While these studies support a role for EV‐miRNA communication in modulating gliotic inflammatory responses, they do not address how intercellular exchange of miRNA by EV occurs and whether such communication pathways are indeed altered in degeneration. Our findings in this work support a role for EV‐miRNA transfer between neurons and glia, with exogenous neuronal miRNA enrichment quantified in Müller glia, but not microglia, following degeneration. Analysis of sequencing data of Müller‐specific DICER KO mice showed upregulation of neuronal miRNA including miR‐124‐3p, and the photoreceptor cluster miRNA miR‐183/96/182 in KO mice, compared to WT controls (Wohl et al., 2017). Further, we showed that both pri‐ and pre‐ forms of these miRNA had no expression in Müller glia indicating that expression, but not production is evident, strengthening an exogenous transfer mechanism. Similar findings were also reported in the brain, with EV inhibition leading to an 80% reduction in astrocytic expression of miR‐124‐3p, accompanied by reduced levels of glutamate uptake and altered astrocyte functioning, while pri‐levels remain unchanged (Men et al., 2019). Findings from this, and previous works have also shown selective enrichment and high expression of neuronal miRNA in retinal EV, with inhibition of EV biogenesis able to impair miR‐124‐3p transfer from photoreceptors to glia in the retina in degeneration (Wooff et al., 2020). It is worth noting however that additional EV fraction stratification prior to sequencing may improve the rigor of the methodology used in this initial study and exclude the possibility of miRNA binding to small protein complexes separate than EV. Additionally, while miR‐124‐3p is widely reported to increase in both human patients and models of neurodegenerations, miR‐124‐3p expression levels within EV have only been loosely explored (Veremeyko et al., 2019), and warrants further investigation.

Understanding the role and functional relevance of EV‐miRNA transfer is further established in this study, with strong evidence to support the transfer of neuronal miRNA to regulate and repress inflammatory gliotic responses. Our results demonstrated that miR‐183/96/182 target genes were primarily localized primarily to Müller glia and were associated with the regulation pathways including cytokine and chemokine inflammation, apoptosis, and cytoskeletal and signalling responses to degeneration. Conversely, we did not observe the same communication pattern to retinal microglia, with decreased expression of miR‐124‐3p and miR‐183/96/182 in microglia found in degeneration, and minimal target genes of these miRNA found in microglia. This result suggests that EV targeting is likely a cell‐specific response, primarily targeting Müller glia to modulate inflammation in retinal degenerations, or that additionally, a loss of EV‐miRNA communication to microglia in degeneration may exacerbate the observed inflammatory phenotype, as reported in other studies (Ponomarev et al., 2011). Inhibition of EV in our previous works (Wooff et al., 2020) has shown that impaired EV transport of cargo including miR‐124‐3p to the inner retina was associated with reduced retinal function, increased levels of photoreceptor cell death and immune cell recruitment and activation (Wooff et al., 2020), supporting the role of EV‐miRNA cargo in modulating homeostatic and inflammatory processes. In the brain, other neuronally‐produced EV‐miRNA including miR‐9‐5p (Li & Zheng, 2023; Veremeyko et al., 2019; Yuan et al., 2021), miR‐181c (Pounders et al., 2022) and miR‐21‐5p (Pounders et al., 2022; Yin et al., 2020); have similarly been found to target glial cells and have associations with inflammatory pathway regulation in neurodegenerations. Further supporting the role of miRNA in gliotic regulation (Chu‐Tan et al., 2018; Kang et al., 2021; Wohl & Reh, 2016; Wohl et al., 2017), studies have demonstrated the impact of miRNA ablation on glial cells, with miRNA ablation in Müller glia resulting in severe gliosis and photoreceptor degeneration (Kang et al., 2021; Wohl et al., 2017), while in cerebellar astrocytes, ablation resulted in a reactive phenotype, functional decline, seizures, degeneration and death (Tao et al., 2011).

Although it is possible that neuronal miRNA may be taken up via glial‐mediated phagocytic mechanisms (Bejarano‐Escobar et al., 2017), lack of EV‐miRNA transfer in EV‐inhibited mice provides strong support for EV‐mediated communication between photoreceptors and glia. Further, work by Kalargyrou et al. (2021) demonstrated that while photoreceptor‐to‐photoreceptor communication was mediated via nanotubes and not EV, photoreceptor‐to‐glia communication was mediated by EV, with glial‐specific uptake of photoreceptor‐derived EV shown in rodent retinas following subretinal and intravitreal delivery methods (Kalargyrou et al., 2021). In the brain, GFP‐tagged neural stem cell EV were found to primarily targeted to astrocytes supporting a mechanism of neuronal‐to‐glial communication mediated by EV even during development (Yoshimura et al., 2018). Our work extends these findings by unveiling a repertoire of receptor ligand interactions that may facilitate the tropism of EV to both Müller glia and potentially microglia in degeneration. A large portion of these interactions involve cellular adhesion mediated by integrins including those identified in this work, (ITGB1, ITGAV, ITGB2), which are recognised to function as powerful modulators of EV homing and tropism (Edelmann & Kima, 2022). It does however remain to be investigated if these alterations are a result of changes in the proteomic decoration of EV membranes or simply reflects a change in the proportionate contribution of each cell type to the total EV release in the retina. Defining these interactions and expanding to other CNS systems is paramount to the development of cell‐targeted therapeutics required to treat neuroinflammatory degenerations where detrimental inflammatory cascades are localized to glia.

Finally, results from this study show early upregulation of EV cargo loading genes in photoreceptors as early as 1 day following degenerative insult, including *Hnrnpa2b1*, a protein involved in specific miRNA loading into EV (Villarroya‐Beltri et al., 2013). We have previously shown that miR‐124‐3p can be translocated across the retina as early as 1 h following PD initiation (Wooff et al., 2023), likely in an attempt to dampen early inflammatory responses and mitigate inflammatory propagation (Wooff et al., 2023), and while we did not investigate timepoints as early as this in this work, we can strongly speculate that EV cargo loading and transport may occur rapidly following degenerative insults across the CNS, while EV release and transport processes seen in the late‐phase of degeneration may occur as a medium to convey their physiological state to glia and regulate gliotic and inflammatory responses occurring in degeneration. Collectively our findings support a mechanism by which miRNA are selectively packaged and delivered between neurons‐to‐glial via EV to regulate degeneration‐induced gliosis.

## 3 CONCLUSION

In conclusion, results from this work provide novel insights into neural‐to‐glial communication axes in the CNS and demonstrate that EV‐miRNA transfer between photoreceptors and Müller glia in the retina is required to dampen inflammatory gliotic responses contributing to retinal degeneration. Further, our work highlights a suite of molecular players that likely shape both the cargo and tropism of retinal EV, providing the catalyst for the development of targeted therapeutics. Taken together, our results suggest that restoring the natural communication system between neurons and glia may prevent aberrant gliotic and inflammatory propagation, ultimately slowing the progression of retinal and neurodegenerations.

## AUTHOR CONTRIBUTIONS

Adrian V. Cioanca, Yvette Wooff and Riccardo Natoli: conceptualization. Adrian V. Cioanca and Yvette Wooff: methodology and data analysis. Adrian V. Cioanca, Yvette Wooff, Riemke Aggio‐Bruce and Rakshanya Sekar: investigation. Adrian V. Cioanca, Yvette Wooff and Riccardo Natoli: writing—draft, review, and editing. Yvette Wooff and Riccardo Natoli: funding acquisition. This research used NCRIS‐enabled Australian Proteome Analysis Facility (APAF) infrastructure.

## CONFLICT OF INTEREST STATEMENT

The authors report no conflict of interest.

## Supporting information

Supporting Information

Supporting Information
