# Supplementary material for: Multiomic integration reveals neuronal‐extracellular vesicle coordination of gliotic responses in degeneration
Source: J Extracell Vesicles. 2023 Dec 11;12(12):12393. doi: 10.1002/jev2.12393 (PMC10714032; doi:10.1002/jev2.12393)
Supplement: Supplementary file 2 — Supporting Information [file JEV2-12-12393-s002.docx]

**Multiomic integration reveals neuronal-extracellular vesicle coordination of gliotic responses in degeneration.**

**Running head:** Extracellular vesicle neuronal-to-glial crosstalk.

Adrian V. Cioanca^†1,2^, Yvette Wooff^†1,2^, Riemke Aggio-Bruce^1,2^, Rakshanya Sekar^1,2^, Catherine Dietrich^1,3^, Riccardo Natoli^1,2^.

**Affiliations:**

^1^Clear Vision Research Group, Eccles Institute of Neuroscience, John Curtin School of Medical Research, College of Health and Medicine, The Australian National University, Acton, ACT 2601

^2^School of Medicine and Psychology, College of Health and Medicine, The Australian National University, Acton, ACT 2601

^3^Peter MacCallum Cancer Centre, Melbourne, VIC, 3052

^†^These authors contributed equally to the work and share first authorship

Corresponding/senior author email: [Riccardo.natoli@anu.edu.au](mailto:Riccardo.natoli@anu.edu.au)

Corresponding author mobile: 0400813132

**Supplementary data:**

*
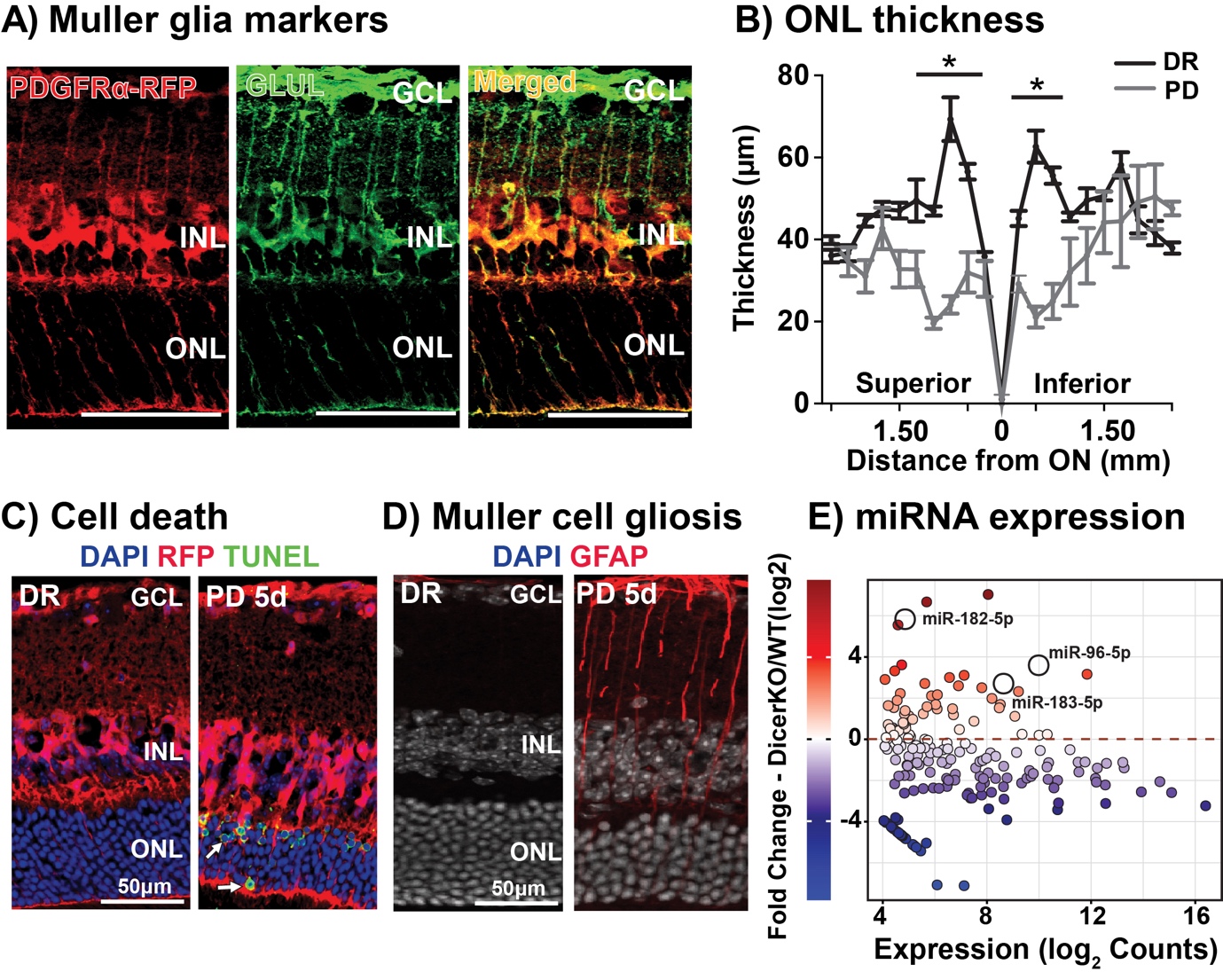
*

**Supplementary Figure 1: PDGFRa-Cre RFP validation and characterization across photo-oxidative damage-induced degeneration. (A)** Müller glia markers to validate PDGFRa-RFP strain showing overlap (orange) between RFP (red) and GLUL (glutamine synthetase; green). **(B)** ONL thickness across the retina in PDGFRa-Cre RFP mice demonstrated significant thinning of the ONL as a response to photo-oxidative damage (P<0.05). **(C)** Increased TUNEL^+^ cells (green) were detected in the ONL following 5d PD indicating increased photoreceptor cell death. **(D)** GFAP labelling (red) was increased in the retina following 5d PD, as a marker of Müller gliosis. **(E)** Volcano plot showing comparative expression of Müller-miRNA compared to in DICER KO- Müller mice. (N=5, P<0.05).


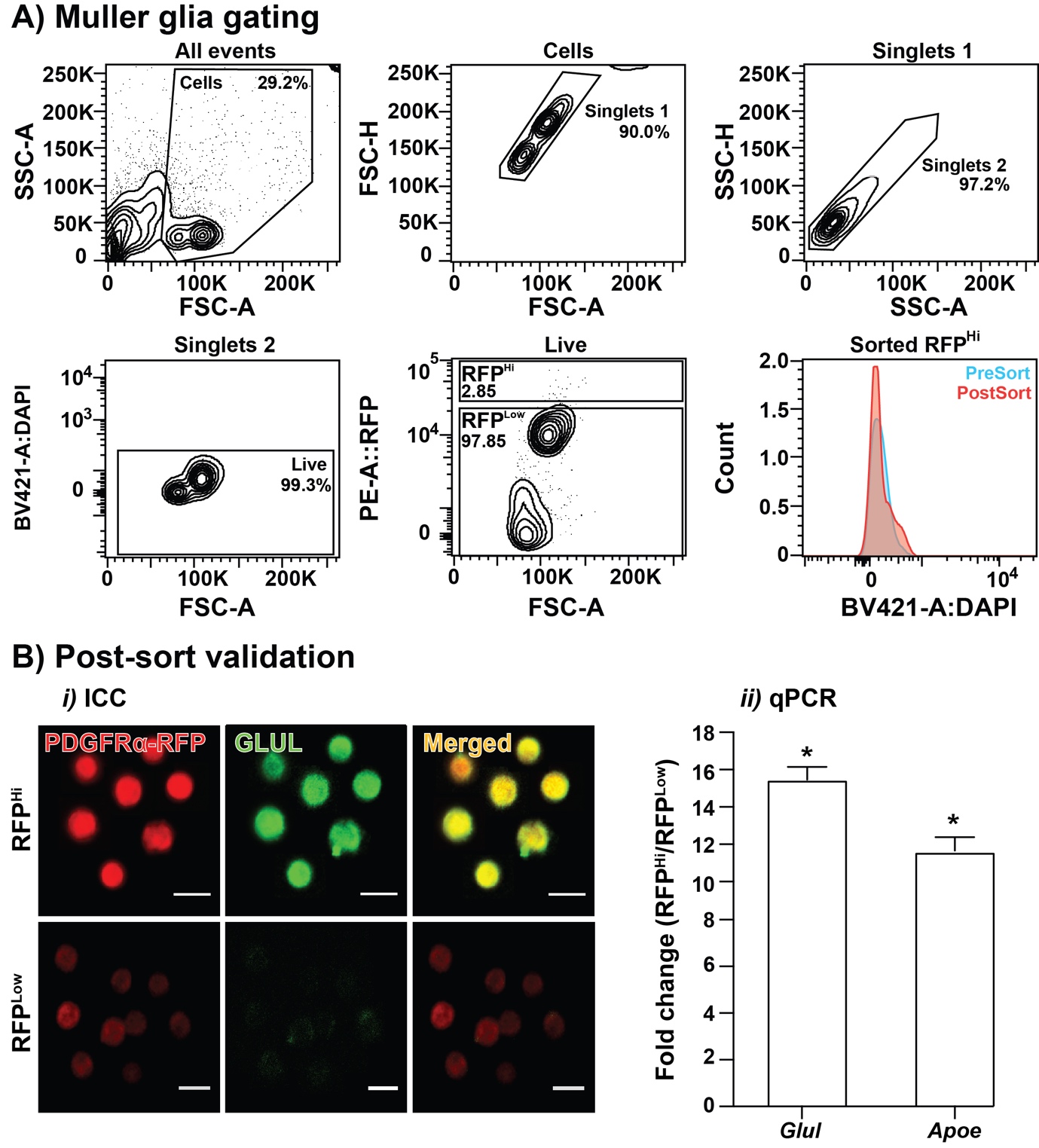


**Supplementary Figure 2: PDGFRa-Cre RFP FACS gating strategy and validation. (A)** FACS gating strategy to isolate RFP^Hi^ populations indicating Müller glia. **(B)** Post-sort validation showing presence of Müller marker GLUL via immunocytochemistry, and increased expression of Müller markers *Glul* and *Apoe* via qRT-PCR in RFP^Hi^ but not RFP^Low^ populations (P<0.05, N=6).


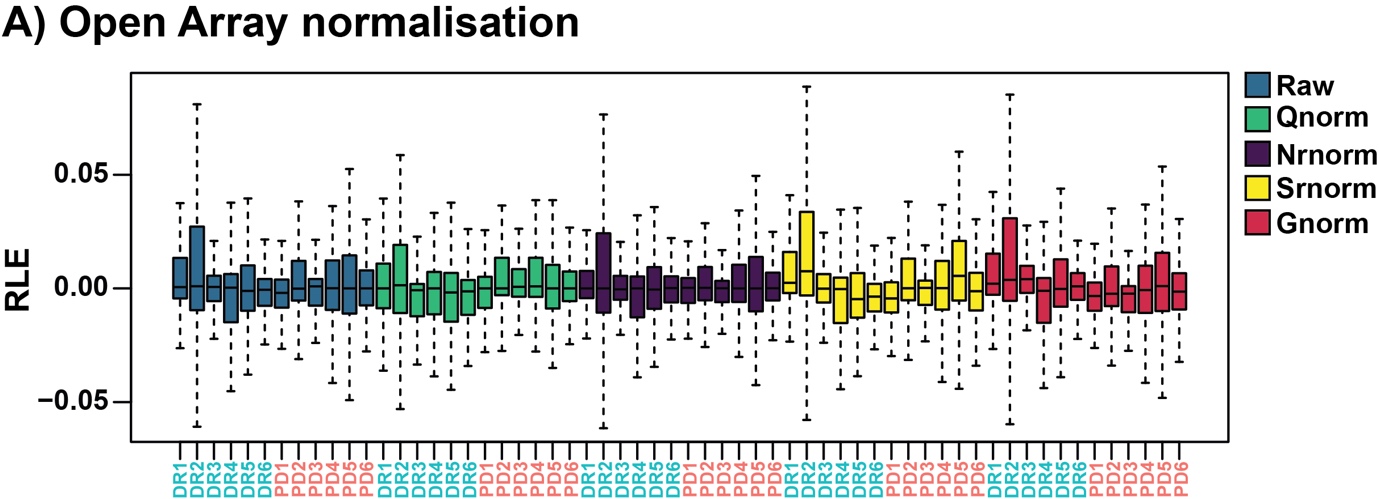


**Supplementary Figure 3: (A)** Methods used for Open Array data normalisation. Qnorm – quantile normalisation; Nnorm – normalisation using rank invariant miRNA; Snorm – normalisation using scale invariant miRNA; Gnorm – normalisation to the geometric mean of each sample. Normalisation using rank invariant miRNA was selected as the preferred method.

**
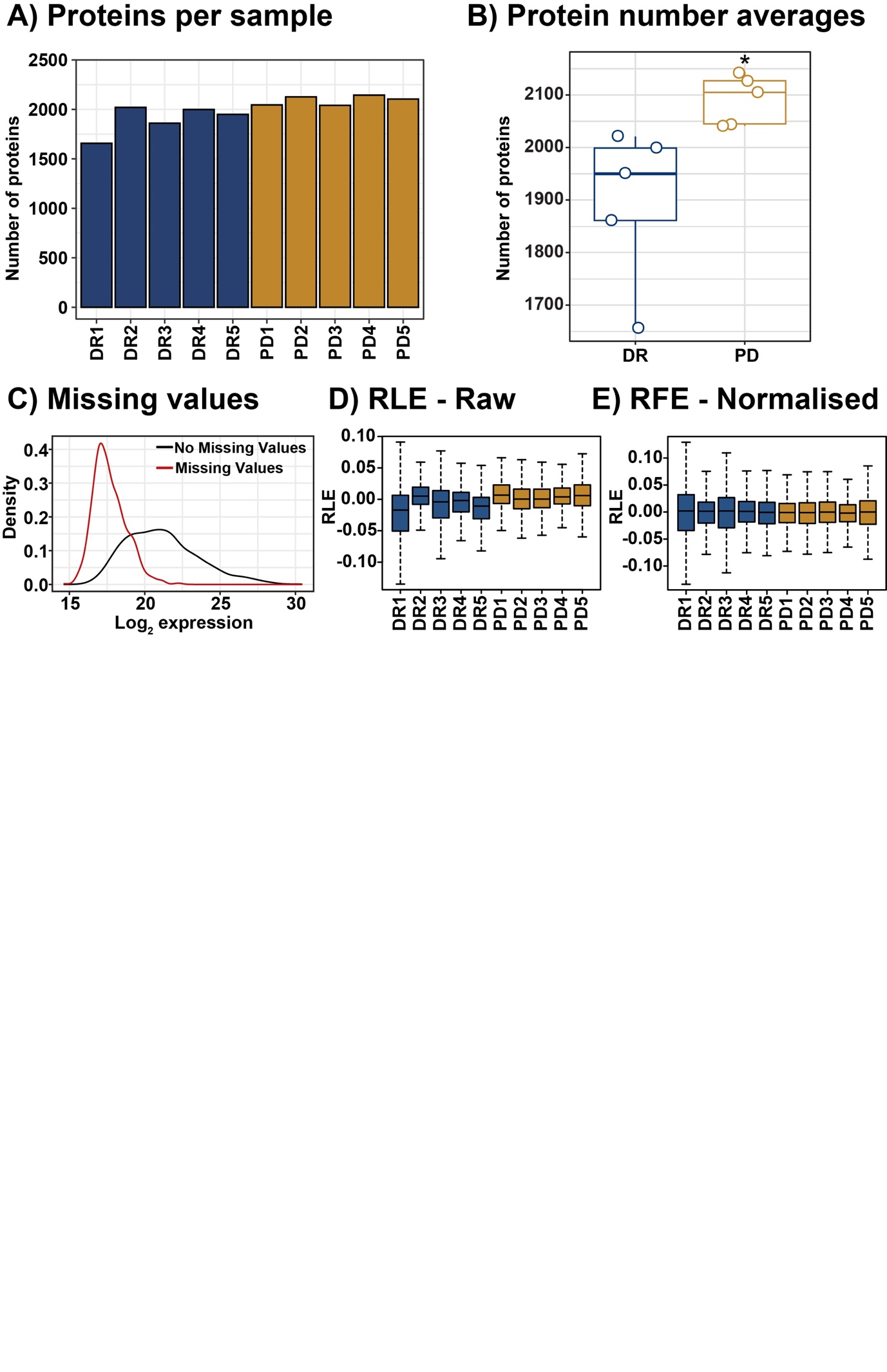
**

**Supplementary Figure 4: Retinal EV protein normalisation. (A)** Number of proteins per sample. **(B)** Average protein number in DR and PD groups. **(C)** Distribution of expression values for proteins with and without missing value. **(D-E)** Raw and normalised relative log expression (RLE) values of EV proteins in DR and PD groups. (N=5).

**
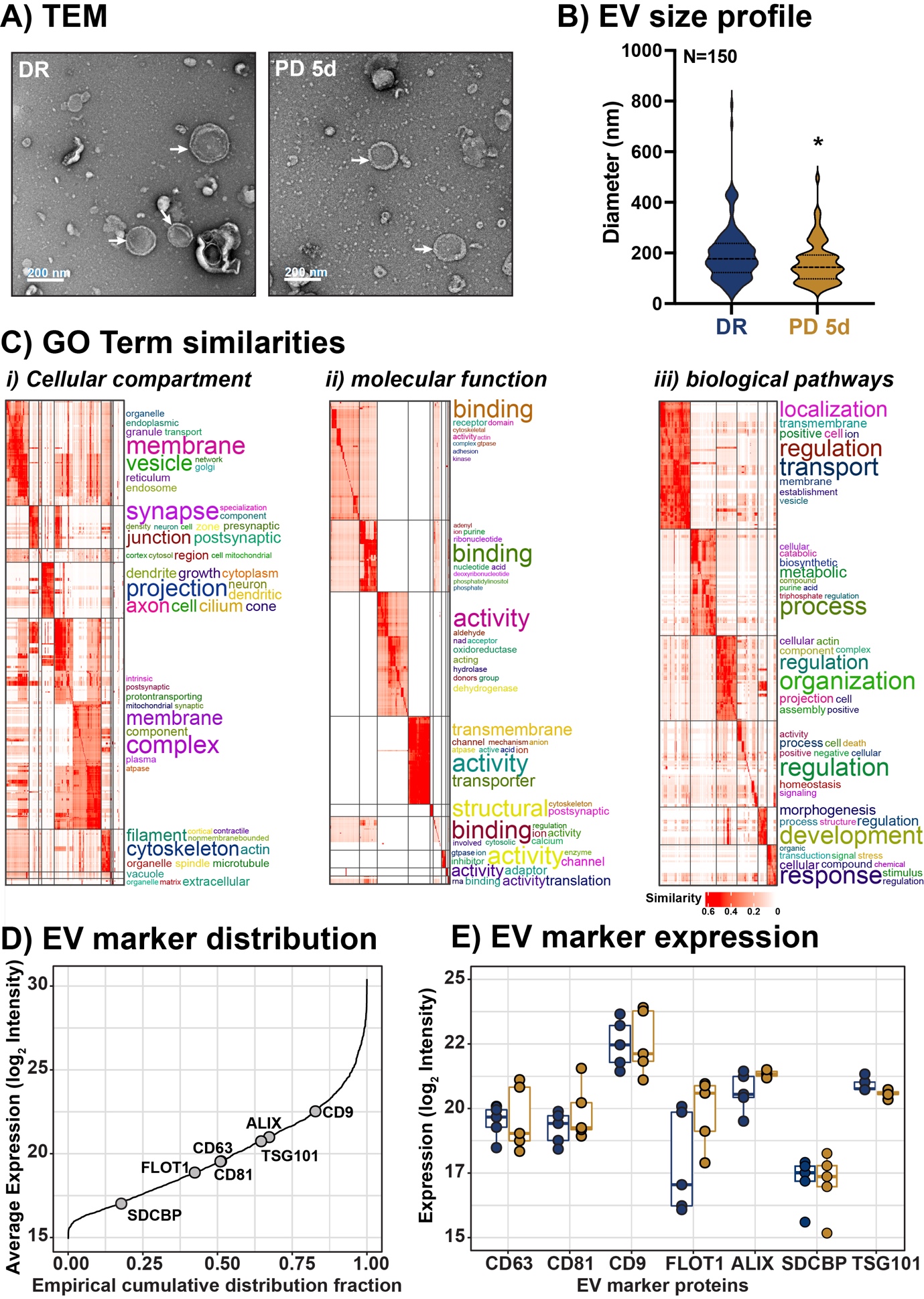
**

**Supplementary Figure 5: Retinal EV characterization profile in DR and PD conditions. (A)** TEM shows EV with cup-shaped rounded morphology. **(B)** EV were found to be significantly smaller following 5d PD. **(C)** GO term analyses of *(i)* cellular compartment, *(ii)* molecular function, and *(iii)* biological pathways using all retinal EV proteins highlight key pathways and processes. **(D)** Expression analyses show enrichment of EV markers across all proteins. **(E)** EV marker expression was unchanged between PD and DR conditions. (N=5).

**
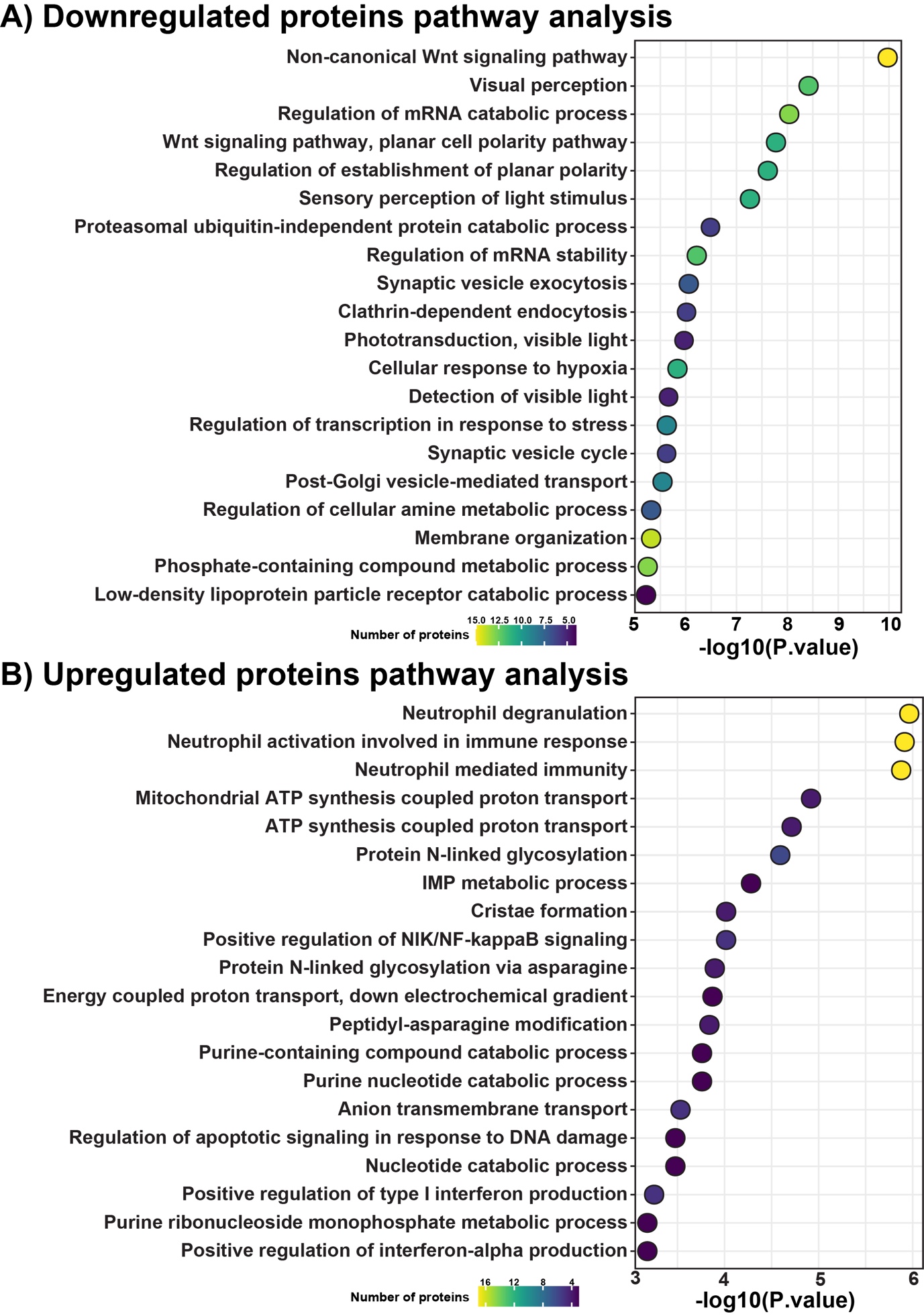
**

**Supplementary Figure 6: Differentially expressed EV proteins involved in homeostatic and immunoregulatory functions.** Pathway analyses of differentially expressed EV proteins showed **(A)** downregulated proteins were involved in homeostatic retinal processes including perception of light, membrane organization, and light detection, while **(B)** upregulated processes were associated with immune regulation and metabolism including immune responses, proton transport, and apoptotic signaling. (N=5, P<0.05).

**
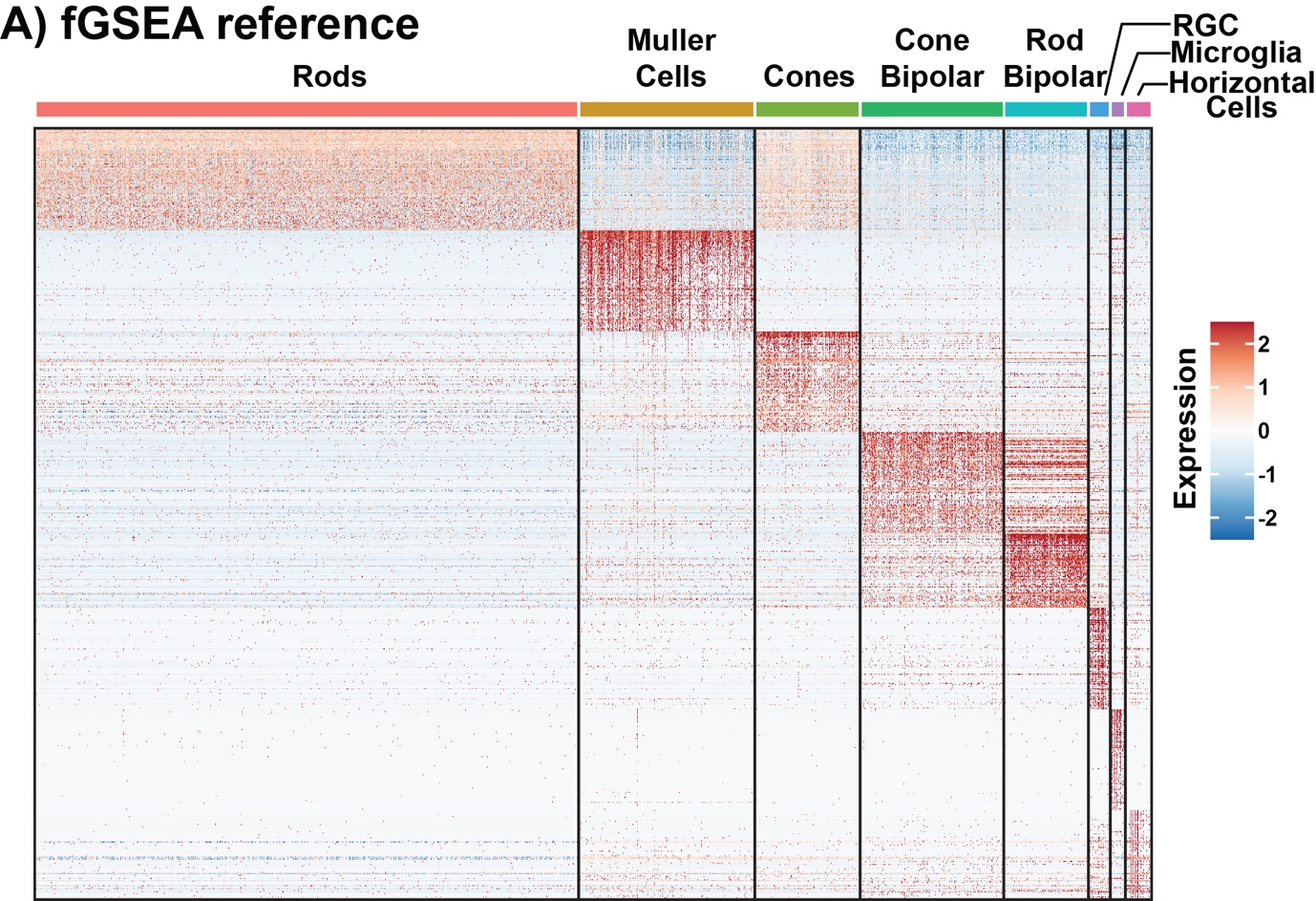
**

**Supplementary Figure 7: Reference fGSEA. (A)** Top 100 differentially expressed genes in major retinal cell types used to generate reference gene sets for gene set enrichment analysis.


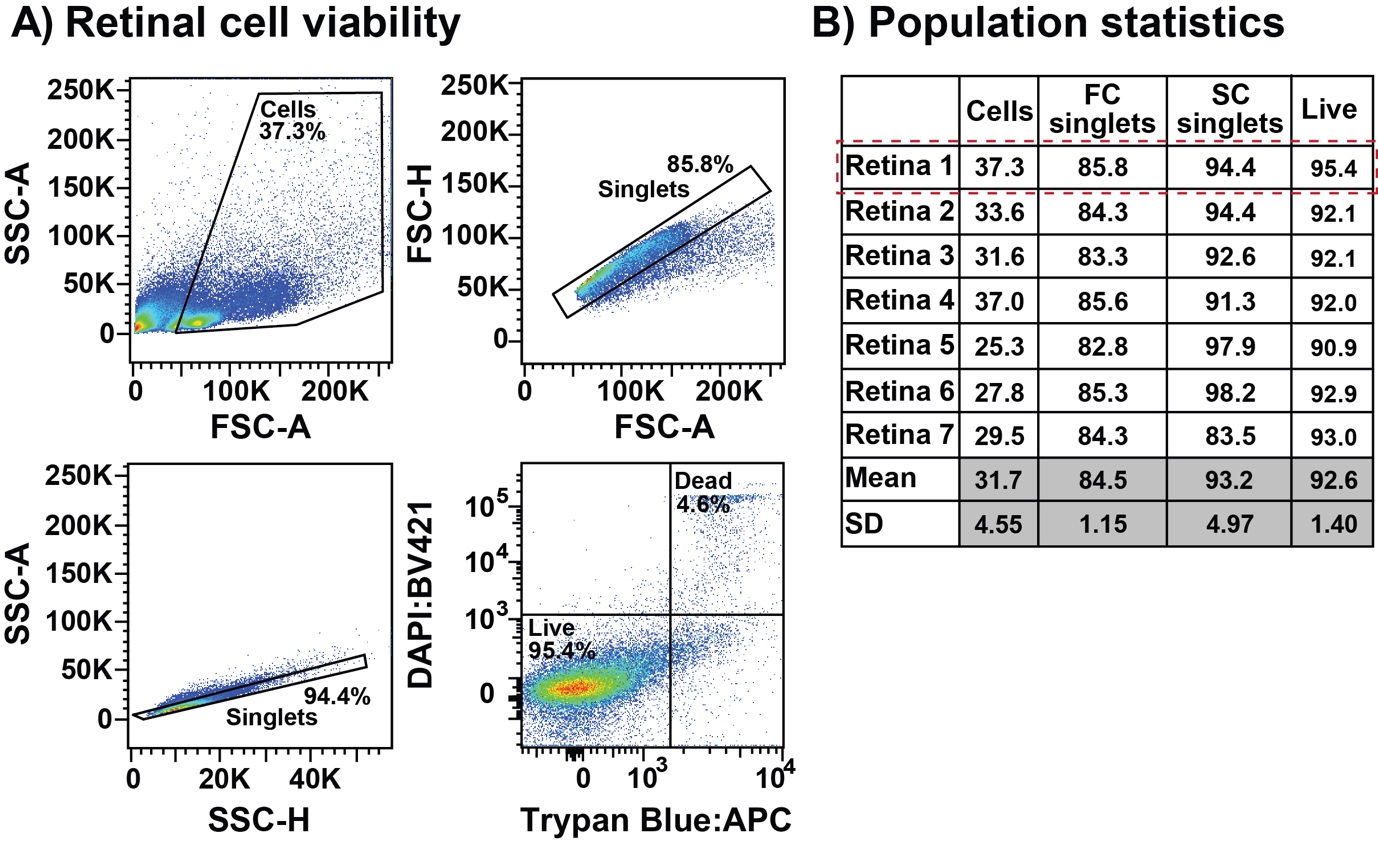
**Supplementary Figure 8: (A-B)** Retinal cell viability following single cell sequencing showing high viability of all samples >90%.


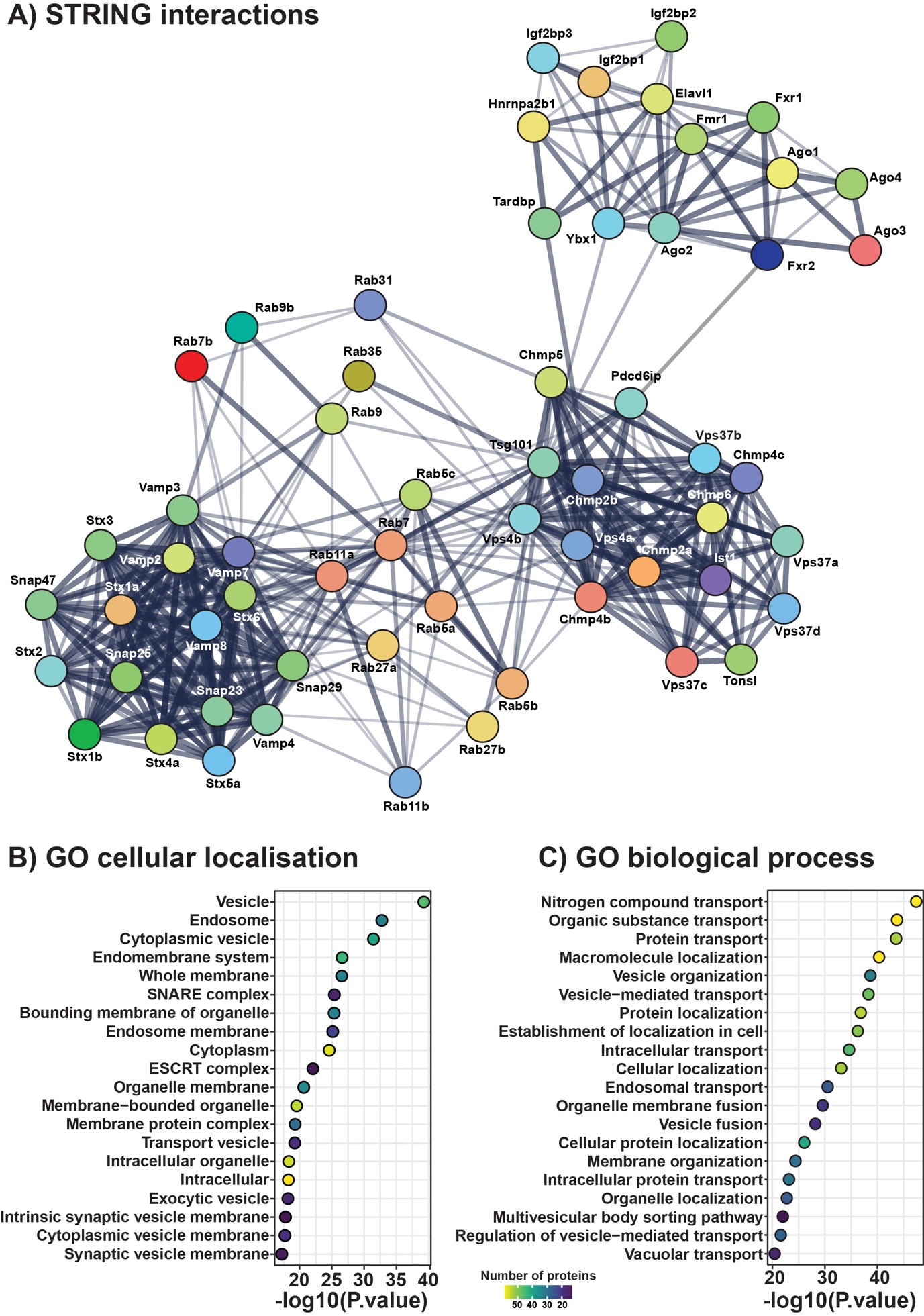


**Supplementary Figure 9: Biological role of genes selected as mediators of EV biogenesis, loading, release, and transport. (A)** STRING interactions showing that genes assembled in a function network. GO term analysis shows the **(B)** cellular localization and **(C)** biological roles of functional network genes.
